# Parenteral dextrose during refeeding is associated with electrolyte deficiencies in anorexia nervosa: a route-specific analysis of oral and parenteral nutrition

**DOI:** 10.1186/s40337-026-01627-5

**Published:** 2026-05-06

**Authors:** Michitaka Funayama, Akihiro Koreki, Yu Mimura, Taketo Takata, Tatsuhiko Yagihashi, Satoyuki Ogino, Shin Kurose, Yusuke Shimizu, Shun Kudo, Akira Nishi, Genki Koyama, Riku Yonezawa, Koki Hosoya

**Affiliations:** 1 Department of Neuropsychiatry, Ashikaga Red Cross Hospital, Ashikaga, Tochigi 326-0843 Japan; 2 Department of Psychiatry, Keio University School of Medicine, Shinjuku, Tokyo Japan; 3 Department of Psychiatry, NHO Shimofusa Psychiatric Medical Center, Chiba-city Chiba, Japan; 4 Jichi Children’s Medical Center Tochigi, Shimotsuke, Tochigi Japan; 5 Department of Trauma and Critical Care Medicine, Kyorin University School of Medicine, Mitaka, Tokyo Japan

**Keywords:** Oral nutrition, Parenteral dextrose, Refeeding syndrome, Electrolyte deficiency, Magnesium, Calcium

## Abstract

**Background:**

Although oral feeding is generally preferred over parenteral nutrition during refeeding in patients with anorexia nervosa, parenteral nutrition often plays a critical role in ensuring adequate nutritional support during early refeeding and preventing underfeeding syndrome. However, few studies have examined route-specific effects on refeeding-related electrolyte deficiencies while accounting for the actual caloric intake delivered via each route.

**Methods:**

We retrospectively examined 208 admissions from 98 patients with anorexia nervosa who were hospitalized in the psychiatric ward of Ashikaga Red Cross Hospital between January 2000 and June 2025. The mean age was 35.3 ± 11.1 years, and the mean body mass index (BMI) at admission was 12.2 ± 2.2 kg/m². In 139 of the 208 admissions (66.8%), nutrition was administered via both oral and parenteral routes. Outcome variables included serum electrolyte levels (phosphorus, potassium, magnesium, and calcium) at admission, at the in-hospital nadir, and the percent decrease from admission to nadir. Explanatory variables included caloric intake via the oral route (with a regular diet and enteral formulas analyzed separately) and the parenteral route (with dextrose and non-dextrose nutrients [amino acids and lipids] analyzed separately), electrolyte provision per calorie, BMI, and admission laboratory data. Multivariable mixed-effects regression analyses were performed.

**Results:**

Caloric intake from a regular diet, enteral formulas, and parenteral non-dextrose administration was not associated with nadir electrolyte levels or with percent decreases from admission. In contrast, higher parenteral dextrose caloric intake was significantly associated with lower nadir magnesium levels (*p* < 0.001) and with greater percent decreases in magnesium and calcium (*p* < 0.001, < 0.05, respectively). Importantly, electrolyte provision per calorie via the parenteral route was not lower than that via the oral route after accounting for reported gastrointestinal absorption rates and carbohydrate proportions in each route.

**Discussion:**

These findings indicate that higher parenteral dextrose administration during refeeding is associated with electrolyte decreases. This likely reflects route-specific differences in glucose handling: parenteral nutrition delivers glucose directly and rapidly into the systemic circulation, bypassing hepatic first-pass uptake that normally buffers systemic glucose and insulin exposure during oral intake. Our findings underscore the need for heightened vigilance for electrolyte deficiencies, particularly when administering parenteral dextrose.

## Abstract (plain language summary)

When individuals with severe anorexia nervosa begin eating again, their bodies can develop dangerous electrolyte deficiencies such as hypophosphatemia and hypomagnesemia. This is a well-known risk during recovery, and careful nutritional management is essential. In this study, we examined whether the way nutrition is given—either by mouth or through the intravenous route—affects this risk. We found that eating food or receiving nutrition through feeding formulas did not increase the likelihood of these deficiencies. However, glucose (sugar) in intravenous nutrition, given directly into a vein, was linked to greater drops in important electrolytes, particularly magnesium and calcium. This difference may be explained by how the body processes nutrients. When food is eaten, it is first handled by the digestive system and the liver, which helps regulate how sugar enters the bloodstream. In contrast, glucose given through a vein bypasses this process and enters the bloodstream rapidly, which may place more strain on the body’s balance. These findings suggest that while intravenous nutrition can be necessary in individuals with severe anorexia nervosa, special attention is needed when giving glucose in this way. Careful monitoring of electrolytes is especially important during this phase of recovery.

## Introduction

Anorexia nervosa is a severe eating disorder characterized by persistent restriction of energy intake, low body weight, and a high risk of medical complications resulting from severe malnutrition. During initial phases of hospitalization for patients with severe anorexia nervosa, refeeding is the cornerstone of medical treatment. Recent evidence supports initiating refeeding in patients with anorexia nervosa using higher-calorie diets (provision of > 1,400 kcal/day) from the outset [1–6]. This approach does not significantly increase the risk of refeeding-related electrolyte deficiencies and helps prevent underfeeding syndrome [1–6], a condition resulting from insufficient caloric provision during the early phase of refeeding in malnourished patients, which is associated with persistent malnutrition, poor weight restoration, delayed recovery, an increased risk of serious medical complications, and even death [7, 8]. Therefore, ensuring adequate caloric intake during refeeding is crucial, particularly in severe anorexia nervosa, while minimizing the risk of clinically significant refeeding syndrome.

Regarding the optimal route for refeeding, several studies have examined the clinical efficacy and safety of oral versus parenteral nutrition in individuals with anorexia nervosa, primarily focusing on complication profiles, although neither actual caloric intake nor electrolyte provision was assessed. Diamanti et al. (2007) [9] compared regular diet feeding alone with combined regular diet and parenteral feeding in patients with anorexia nervosa and found that the parenteral-assisted group achieved greater weekly weight gain and higher maximum caloric intake. However, complications—including infections and elevated transaminases—were significantly more frequent in this group, although all resolved without long-term consequences. Similarly, Michihata et al. (2014) [10] reported that, compared with tube-fed refeeding using enteral formulas, parenteral nutrition alone was associated with a significantly higher incidence of severe complications, such as sepsis and disseminated intravascular coagulation. In contrast, tube feeding with enteral formulas has not been associated with an increased risk of complications compared with a regular diet [11, 12]. Based on these findings, parenteral refeeding is generally not recommended unless no enteral route—including both regular diet and tube feeding with enteral formulas—is feasible [13].

However, clinical reality is often more complex. In patients with severe anorexia nervosa, gastrointestinal complications may preclude oral or tube-fed refeeding. These include superior mesenteric artery syndrome, constipation, intestinal malabsorption, esophageal and gastric dysmotility, lower gastrointestinal motility disorders, and rectoanal motor weakness [14]. Moreover, many patients are unable to consume adequate calories after prolonged malnutrition or continue to engage in binge–purge behaviors [14]. In some patients, nasogastric tubes may be removed by the patients themselves because of discomfort [15]. Under such circumstances, exclusive reliance on a regular diet or tube-fed nutrition may increase the risk of underfeeding syndrome. Parenteral nutrition can therefore play a critical role in ensuring adequate nutritional support during early refeeding and may be used alone or in combination with a regular diet and/or tube feeding in patients with severe malnutrition.

How electrolyte deficiencies during the refeeding period differ by feeding route remains poorly understood, despite their clear clinical importance. Although the following studies did not directly compare oral and parenteral nutrition in the context of anorexia nervosa, historical reports—independent of the anorexia nervosa context—have linked severe refeeding-related electrolyte deficiencies, including lethal hypophosphatemia, to concentrated calories via total parenteral nutrition in malnourished patients [16, 17]. Since then, refeeding-related electrolyte abnormalities have been reported predominantly in patients receiving parenteral nutrition, particularly total parenteral nutrition [18, 19], although they can occur after any form of nutritional reintroduction, including a regular diet, enteral nutrition, or parenteral nutrition [20]. To our knowledge, only two studies have partially addressed this issue through comparative analyses. The first, by Diamanti et al. (2007) [9], as noted above, compared patients with anorexia nervosa receiving a regular diet alone with those receiving a combination of a regular diet and parenteral nutrition and—although not specifically designed for this purpose and without formal statistical testing—reported that electrolyte declines, such as hypophosphatemia and hypokalemia, occurred only in the parenteral-assisted group. In contrast, Zeki et al. [21] reported the opposite pattern, although their cohort did not focus on anorexia nervosa and included individuals without low BMI. Among 92 hospitalized adults at risk for refeeding hypophosphatemia, incidence was significantly higher with tube-fed enteral nutrition than with parenteral nutrition (33% vs. 13%).

However, neither study reported caloric prescriptions, quantified the calories actually delivered by each feeding route, nor assessed electrolyte provision. These limitations are important because glucose delivery via parenteral nutrition is more rapid, prompting insulin surges and subsequent declines in serum electrolytes [22], whereas incretin hormones are released in response to oral nutrient intake but not intravenous nutrition [21]. In addition, carbohydrate proportions and electrolyte composition differ among regular diets, tube-fed enteral formulas, and parenteral nutrition [23–25]. Carbohydrates are theoretically expected to exert a greater impact on electrolyte decline than amino acids and lipids, as refeeding-related electrolyte decreases are thought to result from insulin secretion triggered by carbohydrate intake, leading to intracellular electrolyte shifts [13, 22, 23, 26]. Together, these considerations highlight the need for a comprehensive evaluation of feeding route–specific effects on electrolyte abnormalities during refeeding, with explicit consideration of actual caloric intake by route and electrolyte provision. However, to our knowledge, no previous studies have examined these issues during refeeding in patients with anorexia nervosa.

To address these gaps, we retrospectively examined the effects of a regular diet, enteral formulas, parenteral dextrose, and parenteral non-dextrose nutrients (amino acids and lipids) on electrolyte deficiencies during refeeding in individuals with anorexia nervosa. Reflecting real-world practice, in which feeding routes are often combined and adjusted over time, we quantified route-specific caloric delivery and examined its independent associations with nadir electrolyte levels and percent decreases from admission during the refeeding period. This approach was designed to clarify feeding route–specific contributions to electrolyte deficiencies, a key component of refeeding syndrome.

## Methods

### Participants

The ethical aspects of this retrospective study were reviewed and approved by the Human Research Ethics Committee of Ashikaga Red Cross Hospital (approval number 2025-9). This study was not preregistered. Informed consent was obtained using an opt-out procedure, with detailed information provided on the hospital website. Participants were identified from inpatients admitted to the psychiatric ward of Ashikaga Red Cross Hospital between January 2000 and June 2025. During this period, 208 admissions occurred among 98 patients with anorexia nervosa (ICD-10 code F50.0 [27]) who had a body mass index (BMI) below 17.5 km/m^2^ [28, 29] and were treated in our ward.

Our hospital is the only general hospital in this region, serving approximately 2 million people, with both a tertiary emergency care center and a psychiatric ward capable of managing severe involuntary admissions. Accordingly, our psychiatric ward admits patients with severe psychiatric conditions and serious medical complications, such as severe anorexia nervosa, and manages them with support from specialists in relevant medical fields [30]. Patients with severe anorexia nervosa often present with prominent psychological and/or psychiatric symptoms, limited insight into their physical condition, and may discontinue treatment despite life-threatening malnutrition [31]. Therefore, integrated management of both psychiatric and medical conditions is essential. Our psychiatric ward is structured to provide such combined care, enabling the management of severe anorexia nervosa with complex medical complications. All staff members receive specialized medical emergency response training. We routinely assess patients’ physical condition, particularly malnutrition, using BMI, vital signs, and blood tests at admission, as well as information on food intake immediately before admission. Refeeding is then initiated promptly, and a specialized nutrition support team is consulted to assess nutritional status and determine an appropriate nutritional prescription. This multidisciplinary system allows us to manage severe anorexia nervosa within the psychiatric ward while addressing both medical and psychiatric needs, which frequently coexist in this population.

Patients were classified as having either the restricting type (F50.01, anorexia nervosa, restricting type) or the binge-eating/purging type (F50.02, anorexia nervosa, binge-eating/purging type). Each patient was evaluated by at least two psychiatrists, including at least one board-certified specialist and one psychiatrist with more than 10 years of clinical experience. Among the 208 admissions, subsets of 63, 89, and 101 admissions had been included in our three previous reports [32–34], respectively. Electronic medical records of eligible participants were reviewed retrospectively.

### Caloric delivery and electrolyte provision

We primarily use a regular diet as the route of nutritional support, followed by enteral formulas administered either orally or via tube feeding, whereas parenteral nutrition is reserved as a last option. However, because many of our patients present with a body mass index below 15 kg/m², corresponding to extreme anorexia nervosa [35], and often below 12 kg/m², which places them at markedly increased risk of serious medical complications [35], parenteral nutrition is often used in combination with a regular diet and/or enteral formulas to support refeeding and prevent underfeeding syndrome. In addition, parenteral nutrition may be necessary when nasogastric tubes are removed because of discomfort [15] or when gastrointestinal complications are present [14]. In such cases, when oral feeding is not feasible, peripheral parenteral nutrition is typically initiated from the outset, sometimes in combination with intravenous fluid therapy, while total parenteral nutrition is reserved for the most severe cases. In Japan, parenteral nutrition is frequently administered using glucose–amino acid solutions without lipid emulsions, particularly during short-term nutritional support [36]. Lipid emulsions are typically administered separately, and are often omitted during the early stages of hospitalization. Consequently, carbohydrates account for approximately 70–80% of total caloric intake during early hospitalization, exceeding the proportion typically observed in regular diets or enteral formulas.

The initial regular diet caloric prescription is generally 1,600–1,800 kcal/day. Importantly, the values presented here represent prescribed caloric targets rather than achieved intake. Actual caloric intake was calculated for each admission and is described below. Caloric delivery, unlike electrolyte provision, was not modified in response to electrolyte abnormalities.

Electrolyte provision was managed by the attending physicians on the basis of frequent blood test results [37, 38]. In clinical practice, however, electrolytes were often administered proactively from the outset of treatment, before the development of refeeding syndrome, in line with reports suggesting that early supplementation helps prevent electrolyte deficiencies during the refeeding period [39].

### Data collected

#### Outcome indicators

As outcome indicators, we examined changes in serum electrolyte levels during the refeeding period, focusing on phosphorus, potassium, magnesium, and calcium [22]. For each electrolyte, values were assessed at admission, at the in-hospital nadir, and as percent decreases from admission, calculated as the difference between the admission value and the nadir value divided by the admission value. Nadir levels were evaluated during the refeeding period, defined as spanning from the day of admission through hospital day 20. This timeframe was selected based on evidence that electrolyte levels typically reach their nadir within 3–6 hospital days after admission—for example, at 3.5 ± 3.4 hospital days for potassium [33] and 4.9 ± 4.0 hospital days for phosphorus [32]—and then gradually recover, returning to admission levels by hospital day 20 [40].

A laboratory panel was obtained at admission. For the assessment of nadir levels and percent decreases, subsequent blood samples were collected at 7:30 a.m. before breakfast. For admission levels, data from all 208 admissions were analyzed (Table 1). Nadir levels and percent decreases were analyzed only in admissions in which a nadir electrolyte value could be confirmed—that is, cases in which electrolyte levels exhibited a V-shaped trajectory within the first 20 hospital days after admission, including admissions in which the nadir occurred at admission. Admissions without evidence of a V-shaped trajectory during this period were excluded from nadir-based analyses. These included cases in which patients were discharged before a nadir could be confirmed, most often due to self-discharge or treatment discontinuation at the patient’s request. In addition, some patients were discharged before all four electrolytes demonstrated a V-shaped recovery, because frequent blood testing solely to document complete V-shaped recovery of all electrolytes was not considered clinically warranted in some cases.

Serum calcium and magnesium levels were not adjusted for albumin [41, 42] because albumin was not consistently measured during the refeeding period, precluding reliable calculation of albumin-adjusted nadir values or percent decreases from admission. Instead, serum albumin at admission was included as an explanatory variable in analyses of calcium and magnesium levels at admission, at the nadir, and for percent decreases. Including albumin at admission accounts for baseline physical debilitation independent of BMI, as lower albumin levels reflect poorer general condition and have been shown to be associated with nadir potassium levels irrespective of BMI [33].

#### Explanatory variables

For analyses of electrolyte levels at admission, explanatory variables included demographic characteristics (age, sex, illness duration, and anorexia nervosa subtype—restricting or binge–purge), BMI at admission, and laboratory data at admission (blood urea nitrogen/creatinine [BUN/Cr] ratio, serum albumin, and serum creatinine). For analyses of nadir levels and percent decreases for each electrolyte, explanatory variables additionally included route-specific actual caloric intake per body weight, calculated separately for a regular diet, enteral formulas, parenteral dextrose nutrition, and non-dextrose nutrition including amino acids and lipids; electrolyte provision per total calorie for the corresponding electrolyte; and the admission level of the corresponding electrolyte, as described below.

Inclusion of anorexia nervosa subtype was based on prior evidence that the binge–purge subtype is associated with lower potassium levels [33], whereas the restrictive subtype has been associated with lower hematological values during the refeeding period, reflecting a distinct refeeding-related vulnerability [34]. Lower BMI, calculated as weight in kilograms divided by height in meters squared, has been frequently associated with electrolyte deficiencies during the refeeding period [32, 33, 43]. The BUN/Cr ratio was included because it has been reported to correlate with hypophosphatemia during the refeeding period [22, 40] as an indicator of volume depletion or hemoconcentration, although its reliability for this purpose in individuals with anorexia nervosa may be limited, as it is also influenced by other factors, including protein–energy malnutrition, catabolic states related to starvation, and elevated corticosteroid levels [44, 45]. Serum creatinine, a marker of renal function, was included because of its influence on electrolyte balance. Elevated creatinine levels, reflecting impaired renal function, are associated with increased electrolyte concentrations, such as hyperkalemia [46]. Conversely, during the refeeding period, impaired renal function has also been reported to contribute to electrolyte deficiencies [47]. For electrolyte-specific explanatory variables, diuretic use was included in the analyses of potassium, magnesium, and calcium levels [48–50]; the presence of diarrhea (defined as chart-documented watery stool occurring three or more times per day) was included in the analyses of potassium and magnesium levels [51, 52]; and proton pump inhibitor use was included in the analysis of magnesium levels [53].

Although interactions between magnesium and potassium or calcium have been reported [54, 55], and magnesium is known to influence potassium and calcium homeostasis, magnesium was not included as an explanatory variable when modeling admission levels, nadir levels, or percent decreases of calcium and potassium. This decision was based on the assumption that parallel electrolyte decreases during refeeding—largely driven by insulin-mediated intracellular shifts [22]—would exert a shared and dominant influence, making it difficult to isolate magnesium-specific effects. To address this limitation, we examined correlations among all electrolyte pairs (six combinations across four electrolytes) at admission, at the nadir, and for percent decreases. In particular, correlations between magnesium and potassium or calcium at the nadir were evaluated to identify statistically significant and robust associations in the refeeding context, alongside correlations for the other electrolyte pairs, thereby providing insight into potential magnesium-specific interactions.

### Assessment of route-specific caloric intake

To accurately examine the effect of energy intake relative to body weight, we used actual caloric intake per body weight as the exposure variable. This approach is consistent with dietary therapy in diabetes mellitus [56] and with our previous studies in individuals with anorexia nervosa [32–34], although those studies did not distinguish route-specific energy intake. In the present study, actual caloric intake (kilocalories) was defined as the mean total intake over the first seven hospital days [32–34, 40] and was calculated separately for a regular diet, enteral formulas, parenteral dextrose, and parenteral non-dextrose components.

Although non-oral nutrition in our cohort consisted of a mixture of intravenous fluid therapy, peripheral parenteral nutrition, and total parenteral nutrition, these sources were subcategorized into dextrose and non-dextrose components, the latter including amino acids and lipids. Because the insulin surge responsible for refeeding-related electrolyte shifts is primarily driven by carbohydrate intake—particularly dextrose [13, 22, 23, 26]—it is reasonable to categorize intravenous fluids and parenteral macronutrients according to the presence or absence of dextrose. In addition, the amount of each component could be quantified precisely.

In contrast, estimating actual caloric intake from regular diets and enteral formulas consumed orally rather than via tube feeding requires adjustment because most participants did not consume the entire meal provided. Intake was estimated from nursing records in which food consumption at each meal was documented using a 10-point scale (0 = none, 10 = all). In our hospital, nursing staff are trained to estimate the overall proportion of each meal consumed using this scale, which reflects the entire meal rather than individual components. Food intake was recorded three times daily, yielding a total of 21 meals during the first seven hospital days. For example, if a patient consumed half of a prescribed 1,800-kcal diet (mean score of 5 across the 21 meals), oral intake was calculated as 900 kcal. Subcategorizing a regular diet into dextrose and non-dextrose components is challenging. However, although the consumed portion of each meal could not be partitioned into specific components (e.g., soup, meat, or noodles), the standard hospital diet provides a relatively stable macronutrient composition—approximately 60% carbohydrates, 15% protein, and 25% lipids of total caloric intake—regardless of whether meals are regular or softened.

When patients received enteral formulas, either orally or via tube feeding, their caloric intake was calculated using the same method. Although small differences existed among products, the macronutrient composition of the enteral formulas was approximately 50–60% carbohydrates, 10–20% amino acids, and 25–40% lipids. To estimate the macronutrient proportions for the overall cohort, we calculated weighted averages based on the specific formula used and the amount consumed.

In our ward, enteral formulas were administered mainly orally and occasionally via tube feeding, and some patients receive a combination of a regular diet and enteral formulas. Accordingly, when dichotomizing routes into non-parenteral and parenteral, we used “oral route,” as most non-parenteral intake was oral and “enteral” may imply tube feeding. In addition, tube feeding via a nasogastric tube is provided only as bolus feeds, typically three times per day, and is never administered as continuous feeding. When enteral formulas are consumed orally, they are usually taken once or twice per day as a supplement to the regular diet, although in some cases they are taken three times per day as a replacement for regular meals.

### Assessment of electrolyte provision

Electrolyte provision from both oral and parenteral routes was quantified. We used electrolyte provision during the first 24 h after admission—before refeeding-related electrolyte shifts typically begin—rather than provision over the first seven hospital days, for methodological reasons. Electrolyte supplementation during hospitalization is generally adjusted directly in response to laboratory results; therefore, including electrolyte provision across the entire refeeding period as an explanatory variable could introduce spurious associations with nadir levels or percent decreases, resulting in biased regression estimates due to over-adjustment rather than appropriate confounding control. Nevertheless, early electrolyte provision during refeeding can influence subsequent nadir levels and percent decreases. To address this issue, we included only electrolyte provision during the first 24 h after admission, prior to electrolyte adjustments based on in-hospital blood test results detecting refeeding-related deficiencies.

For each electrolyte, oral and parenteral provision during the first 24 h after admission was calculated separately. Parenteral electrolyte provision was directly calculable. Oral electrolyte provision was determined by summing electrolytes derived from a regular diet, enteral formulas, and oral electrolyte medications. Actual oral electrolyte provision was estimated using the same method described above for actual oral caloric intake, namely, applying the proportion consumed to standardized meal types (e.g., 1,800-kcal regular meals and 1,600-kcal softened meals) and/or enteral formulas, each of which has a nearly fixed electrolyte content. The same approach was applied to enteral formulas. Medication-type electrolyte supplements, including potassium L-aspartate, were included. Magnesium oxide—sometimes prescribed for constipation rather than electrolyte replacement—was also counted as magnesium provision. For reference, we also examined oral and parenteral electrolyte provision for each electrolyte over the first seven hospital days, allowing comparison of electrolyte provision per calorie during the first 24 h with that during the first seven days, to assess whether electrolyte provision per calorie was adequate from the outset.

Of note, unlike orally digested macronutrients such as glucose, which are absorbed efficiently in the gastrointestinal tract, the absorption of orally administered electrolytes varies substantially [57–69] (see note in Table 1). To facilitate comparison of electrolyte provision per calorie across feeding routes, parenteral electrolyte provision per calorie was additionally expressed as a percentage relative to oral provision, allowing direct comparison of route-specific electrolyte provision independent of absolute intake. In Table 1, individual absolute values of oral electrolyte provision are presented without adjustment for gastrointestinal absorption. However, for use as explanatory variables in statistical analyses, oral electrolyte provision was adjusted for gastrointestinal absorption by applying representative absorption rates [57–69] (see notes in Tables 4, 5, 6 and 7 for electrolyte-specific absorption rates).

Ideally, electrolyte provision from each nutritional route would be expressed separately as electrolyte provision per calorie for oral and parenteral nutrition to be used as explanatory variables. However, in 66 of 208 admissions, patients did not receive parenteral nutrition, resulting in zero parenteral caloric intake and precluding calculation of parenteral electrolyte-per-calorie values. Similarly, in 26 admissions, no oral calories were consumed during the first 24 h, rendering oral electrolyte-per-calorie values unavailable. Importantly, restricting analyses to admissions with nonzero caloric intake for both routes would not only lead to division-by-zero problems but would also necessitate exclusion of the most severely ill patients—such as those unable to consume any oral nutrition—thereby introducing selection bias and potentially distorting the clinical interpretation of the results. Therefore, for the primary analysis, we used total electrolyte provision from both nutritional routes during the first 24 h—including electrolytes naturally contained in nutrition and those administered as supplementation—normalized by total caloric intake from both routes during the same period as the explanatory variable.

To mitigate this limitation, we conducted route-specific analyses restricted to oral and parenteral feeding cohorts, defined as admissions in which nutrition during the first 24 h after admission was provided exclusively via one route. These analyses were performed as supplementary investigations where feasible despite limited sample size and examined associations between route-specific electrolyte provision per calorie and the corresponding electrolyte nadir level or percent decrease, as described below.

### Statistical analysis

#### Simple correlation analysis

To obtain an overall picture and to assess whether the direction of associations was consistent with subsequent regression models, we first conducted simple correlation analyses examining relationships between caloric intake or electrolyte provision and refeeding-related electrolyte deficiencies. These analyses were performed in three groups: the entire cohort, a subgroup receiving oral intake only, and a subgroup receiving parenteral intake only, with the latter two defined based on intake during the first 24 h after admission, allowing calculation of route-specific electrolyte-per-calorie values. Because the sample sizes of the route-specific subgroups were insufficient for multivariable regression modeling, these analyses were conducted as supplementary analyses. For each group, correlations were calculated between caloric intake per body weight and both the nadir level and percent decrease of each electrolyte, as well as between electrolyte provision per calorie during the first 24 h and the corresponding nadir level and percent decrease. For the entire cohort, combined electrolyte provision from both oral and parenteral routes was used, whereas, by definition, for the route-specific subgroups, only route-specific electrolyte provision was examined. In these simple correlation analyses, we used the combined caloric intake from a regular diet and enteral formulas to avoid unnecessary complexity. These components were analyzed separately in the following multivariable mixed-effects models.

### Multivariable mixed-effects regression

To comprehensively investigate factors associated with electrolyte levels at admission, at the nadir, and with percent decreases, we used multivariable mixed-effects regression models to account for within-individual clustering due to repeated admissions, with individuals included as random intercepts. Fixed effects included demographic variables (age, sex, illness duration, and anorexia nervosa subtype), BMI, and laboratory data at admission (blood urea nitrogen/creatinine [BUN/Cr] ratio, albumin, and creatinine). For analyses of nadir levels and percent decreases, additional fixed effects included caloric intake per body weight during the first seven hospital days from a regular diet, enteral formulas, parenteral dextrose nutrition, and parenteral non-dextrose nutrition (each analyzed separately); total electrolyte provision per calorie during the first 24 h; and the corresponding electrolyte level at admission. Given the large number of explanatory variables, factors associated with individual electrolytes—such as diuretic use (potassium, magnesium, calcium), proton pump inhibitor use (magnesium), and diarrhea (potassium, magnesium)—were included in the multivariable mixed-effects regression models only when they were significantly associated with the respective electrolytes in simple regression analyses. No numeric explanatory variable showed a correlation coefficient exceeding 0.65 with any other variable. The strongest correlations were observed between age and illness duration (*r* = 0.64), age and albumin at admission (*r* = − 0.57), and albumin and calcium at admission (*r* = 0.50), indicating limited multicollinearity and supporting retention of all variables in the models.

### Multilevel correlation analyses among electrolyte levels

Correlations among electrolyte levels at admission, at the nadir, and for percent decreases were examined using multilevel correlation analyses to account for within-individual clustering due to repeated admissions.

### Multiple-comparison adjustment

Multiple comparisons were adjusted using the Bonferroni method. For mixed-effects regression models, an adjustment factor of four was applied, corresponding to the four electrolytes analyzed. For multilevel correlation analyses, an adjustment factor of six was applied, reflecting the six pairwise combinations among the four electrolytes. Unadjusted estimates from mixed-effects models for each electrolyte were also presented to facilitate electrolyte-specific interpretation and to convey the magnitude and direction of effects, particularly for oral and parenteral caloric intake. Similarly, unadjusted simple correlation analyses were performed to illustrate the direction of associations and were not subjected to multiple-comparison adjustment. Two-tailed p values < 0.05 were considered statistically significant. All analyses were conducted using R (version 4.1.1).

## Results

All patients underwent laboratory testing at admission and were frequently monitored with serial testing thereafter. A second laboratory panel was obtained by hospital day 4 in 164 admissions (78.8%). Specifically, blood tests were performed in 135 admissions (64.9%) on hospital day 2, 123 (59.1%) on day 3, 121 (58.2%) on day 4, 107 (51.4%) on day 5, 80 (38.5%) on day 6, 75 (36.1%) on day 7, 76 (36.5%) on day 8, 50 (24.0%) on day 9, and 64 (30.8%) on day 10.

**Fig. 1:**
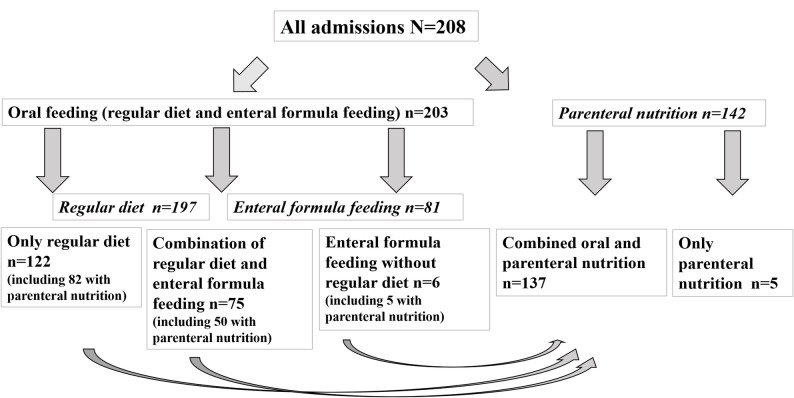
Distribution of nutritional routes during the first 7 hospital days

Figure 1 illustrates the distribution of nutritional routes among the 208 admissions. During the first seven hospital days, oral feeding—including both regular diet and enteral formulas—was implemented in 203 of 208 admissions (97.6%). The five admissions involved severe physical conditions that precluded oral feeding. Among the 81 admissions receiving enteral formulas, 10 (4.8% of total admissions) received them via nasogastric tube feeding, while the remaining 71 consumed them orally. Among the 81 admissions receiving enteral formulas, 75 also received nutrition from a regular diet. Parenteral nutrition was administered in 142 admissions (68.3%), of which 137 (65.9%) also received oral feeding. Among these, total parenteral nutrition was provided in 55 admissions (26.4%), including 54 admissions (26.0%) in which it was combined with oral feeding. Overall, 137 admissions (65.9%) received combined oral and parenteral nutrition, and 54 admissions (26.0%) received combined oral and total parenteral nutrition.

During the first 7 hospital days, total caloric intake averaged 1443 kcal/day, including 1109 kcal/day from oral nutrition—comprising approximately 942 kcal/day from regular diets (85%) and 167 kcal/day from enteral formulas (15%)—and 334 kcal/day from parenteral nutrition (Table 1). Macronutrient composition was similar between the regular diet and enteral formulas; however, parenteral nutrition was characterized by a low lipid and high carbohydrate proportion. Specifically, carbohydrate proportions were approximately 60%, 54%, and 81% for the regular diet, enteral formulas, and parenteral nutrition, respectively. The corresponding proportions of amino acids were 15%, 13.8%, and 15%, and those of lipids were 25%, 32.2%, and 4%, respectively.

Table 1 summarizes demographic characteristics and clinical data for admissions with anorexia nervosa. The leftmost column presents data from all 208 admissions (98 individuals), which were used for analyses of electrolyte levels at admission. The mean BMI across all 208 admissions was 12.2 ± 2.2, indicating that the cohort predominantly comprised individuals with extreme malnutrition [35]. For multivariable mixed-effects regression analyses at admission, complete explanatory data were available for 187 admissions for phosphorus, 192 for potassium, 150 for magnesium, and 174 for calcium after excluding admissions with missing variables, such as individual electrolyte measurements or serum albumin levels. The relatively smaller number of admissions with available magnesium data reflects historical limitations in laboratory testing at our hospital. During the first three years of this 25-year study period, rapid serum magnesium testing was unavailable and result reporting required several hours. Consequently, magnesium measurement at admission was sometimes omitted at that time, particularly in patients with less severe malnutrition (e.g., BMI around 16), although it was frequently measured during hospitalization. The four rightmost columns present data used for analyses of nadir levels and percent decreases for each electrolyte: serum phosphorus (172 admissions from 84 individuals), potassium (182 admissions from 88 individuals), magnesium (140 admissions from 62 individuals), and calcium (148 admissions from 75 individuals). Admissions without evidence of a V-shaped trajectory during this period were excluded from nadir-based analyses (15 admissions for phosphorus, 10 for potassium, 10 for magnesium, and 26 for calcium).

Regarding electrolyte provision (Table 1), absolute oral electrolyte provision was derived primarily from regular diet and enteral formulas. Medication-type supplementation accounted for roughly 12% of total oral potassium provision and 45% of total oral magnesium provision (the latter largely in the form of magnesium oxide, which was also prescribed for constipation), whereas it contributed minimally to phosphorus (≈ 0.5%) and not at all to calcium (0.0%). In terms of electrolytes per calorie, enteral formulas contained comparable or slightly higher electrolyte levels than the regular diet, with phosphorus approximately 1.5-fold higher, comparable for potassium, 1.3-fold higher for magnesium, and 1.6-fold higher for calcium. With respect to route-specific differences, parenteral electrolyte provision per calorie was substantially higher than oral provision for phosphorus, potassium, and magnesium and was comparable for calcium during both the first 24 h and the first seven hospital days after admission. These comparisons accounted for reported gastrointestinal absorption rates of orally administered electrolytes [56–68] and for the higher carbohydrate proportion in parenteral nutrition (81%) than in regular diets and enteral formulas (60% and 54%, respectively), which may induce an insulin surge approximately 1.3–1.5 times greater. In addition, parenteral electrolyte provision per calorie during the first 24 h after admission was higher than or comparable to that during the first seven hospital days, indicating that parenteral electrolyte provision per calorie was not low at the initiation of refeeding.

Electrolyte deficiencies were highly prevalent. Hypophosphatemia (< 2.5 mg/dl) was observed in 72 of 172 admissions (41.9%) at some point during hospitalization. Hypokalemia (< 3.5 mEq/l) occurred in 120 of 182 admissions (65.9%), hypomagnesemia (< 1.7 mg/dl) in 64 of 140 admissions (45.7%), and hypocalcemia (< 8.5 mg/dl) in 100 of 148 admissions (67.6%). Mean serum phosphorus decreased from 3.7 ± 1.0 mg/dl at admission to 2.6 ± 0.8 mg/dl (− 29.7%), with the nadir observed on hospital day 5.1 ± 4.5. Mean serum potassium decreased from 3.6 ± 1.0 mEq/l to 3.1 ± 0.7 mEq/l (− 13.9%) on hospital day 4.1 ± 4.3. Mean serum magnesium decreased from 2.3 ± 0.7 mg/dl to 1.8 ± 0.3 mg/dl (− 21.7%) on hospital day 6.4 ± 5.2. Mean serum calcium decreased from 8.9 ± 1.2 mg/dl to 8.0 ± 0.9 mg/dl (− 10.1%) on hospital day 5.9 ± 4.7.

Despite this mean timing, potassium frequently reached its nadir early: 98 admissions (53.8%) occurred by hospital day 2. In contrast, fewer nadirs of the other electrolytes were observed by hospital day 2: phosphorus in 50 admissions (29.1%), magnesium in 44 admissions (31.4%), and calcium in 44 admissions (29.7%). Among admissions in which potassium was already at nadir at admission (69 cases, 37.9%), the binge–purge subtype accounted for more than half (36 cases) and was significantly more frequent than the restrictive subtype when compared with admissions reaching potassium nadir on hospital day 2 or later (chi-square test, *p* = 0.01).

### Simple correlation analysis between electrolyte levels and caloric intake or electrolyte provision

During the first 24 h, the oral group received only oral intake and no parenteral nutrition (~ 1207 vs. ~ 0 kcal/day), whereas the parenteral group showed the opposite pattern (~ 0 vs. ~ 378 kcal/day), with the overall cohort lying between these extremes (~ 923 vs. ~ 251 kcal/day). Although these groups were not strictly exclusive thereafter and some patients also received nutrition via the alternative route, during the first 7 days the predominantly oral group had higher oral and lower parenteral intake (~ 1366 vs. ~ 122 kcal/day), whereas the predominantly parenteral group had lower oral and higher parenteral intake (~ 480 vs. ~ 580 kcal/day), with the overall cohort lying between these extremes (~ 1100 vs. ~ 377 kcal/day), supporting the validity of this subgroup classification. To provide an overall picture of the direction of associations, Table 2 presents simple correlations between electrolyte levels and caloric intake or electrolyte provision, without adjustment for multiple comparisons. The numbers of admissions available for each cohort and each electrolyte are shown in the leftmost column.

In the overall cohort (upper section), higher caloric intake during the first seven hospital days was associated with lower nadir phosphorus and magnesium levels and with higher nadir potassium levels. Among admissions receiving calories predominantly via the oral route from regular diet and enteral formulas (middle section), higher oral caloric intake was associated with lower nadir magnesium levels. In contrast, for the other electrolytes, correlation coefficients were consistently positive, indicating a tendency for higher oral caloric intake to be associated with higher nadir levels, although these associations did not reach statistical significance. Although the number of admissions receiving calories predominantly via the parenteral route was small (bottom section), the observed pattern was opposite: higher parenteral caloric intake showed a consistent directional tendency toward lower nadir levels across all electrolytes, with calcium demonstrating a statistically significant association. Notably, after accounting for the reported gastrointestinal absorption rates of orally administered electrolytes (see note in Table 1) and the higher carbohydrate proportion in parenteral nutrition than in regular diets and enteral formulas, parenteral electrolyte provision per calorie in this group was higher than or comparable to that in the oral group: 155.7% for phosphorus, 128.1% for potassium, and 48.3% for calcium. In contrast, magnesium provision per calorie via the parenteral route was slightly lower than that in the oral group (33.1%).

Regarding electrolyte provision, greater potassium provision during the first 24 h after admission was associated with lower nadir potassium levels in both the overall cohort and the oral group. In contrast, calcium provision during the first 24 h was associated with a smaller percent decrease in serum calcium in the overall cohort and the parenteral group.

### Factors associated with electrolyte levels: multivariable mixed-effects regression

Explanatory variables associated with individual electrolytes, such as diuretic use, proton pump inhibitor use, and the presence of diarrhea, were not associated with their respective electrolytes in the simple regression analyses (*p* > 0.33 for all). Therefore, these variables were not included in the multivariable mixed-effects regression model. Table 3 summarizes factors significantly associated with electrolyte levels identified using multivariable mixed-effects regression models with Bonferroni adjustment. Overall directional tendencies by route-specific nutrition are presented in Table 4 (with Bonferroni adjustment), while the magnitudes of associations without Bonferroni adjustment for all explanatory variables are shown in Tables 5, 6, 7 and 8 for phosphorus, potassium, magnesium, and calcium, respectively.

Caloric intake from a regular diet, enteral formulas, and parenteral non-dextrose nutrients was not associated with electrolyte nadir levels or percent decreases. In contrast, higher parenteral dextrose intake was associated with lower nadir magnesium and greater percent decreases in magnesium and calcium during refeeding (Table 4).

Among covariates other than caloric intake, higher serum creatinine levels at admission were associated with higher electrolyte levels at admission, but with lower nadir levels and greater percent decreases across all electrolytes examined. Lower BMI was associated with lower nadir phosphorus, potassium, and calcium levels and with greater percent decreases in potassium and calcium; it was also associated with lower calcium levels at admission. A higher BUN/Cr ratio was associated with higher potassium levels at admission but with lower nadir levels in phosphorus and greater percent decreases in phosphorus and potassium. The binge–purge subtype was associated with lower potassium levels at admission. Higher serum albumin levels were associated with higher calcium levels at both admission and nadir as well as higher magnesium levels at admission. Across all electrolytes, higher admission levels were consistently associated with higher nadir levels and greater percent decreases for the corresponding electrolyte. With respect to electrolyte provision, higher calcium provision during the first 24 h was associated with higher calcium nadir levels and a smaller percent decrease in calcium. In contrast, the association between potassium provision during the first 24 h and potassium nadir levels observed in simple correlation analyses was not significant in the multivariable mixed-effects regression model.

As shown in Table 4, regardless of statistical significance, higher parenteral dextrose intake showed a consistent directional association with lower nadir electrolyte levels across all electrolytes, consistent with the simple correlation analyses in the parenteral group. In contrast, associations for a regular diet, enteral formulas, and parenteral non-dextrose intake varied, with higher nadir potassium levels across all three routes. Similarly, higher parenteral dextrose intake was associated with greater percent decreases across all electrolytes, whereas associations for the other routes varied, with smaller percent decreases in potassium across all three routes.

### Correlations among electrolyte levels

Multilevel correlation analyses included 146 admissions for admission levels, 121 for nadir levels, and 112 for percent decreases, all with complete data. Table 9 summarizes the results of the multilevel correlation analyses after Bonferroni adjustment.

Overall, associations between magnesium and potassium or calcium were not prominent. At admission, magnesium and calcium levels showed a weak positive correlation (*r* = 0.30), whereas magnesium and potassium levels were not significantly correlated. At nadir, magnesium showed no significant correlation with either potassium or calcium. For percent decreases, weak positive correlations were observed for magnesium–calcium pairs (*r* = 0.34). All electrolyte pairs showed positive directional associations at nadir and for percent decreases, regardless of statistical significance.

**Table 1:** Demographic characteristics, laboratory data, and treatment indicators

Characteristic		Admission electrolyte levels (*N* = 208)	Nadir phosphorus (*n* = 172)	Nadir potassium (*n* = 182)	Nadir magnesium (*n* = 140)	Nadir calcium (*n* = 148)
Demographics	Age (years)	35.5 ± 11.1	35.8 ± 11.0	35.7 ± 11.0	37.0 ± 11.1	36.4 ± 11.0
	Female sex	97.1%	97.7%	97.3%	98.6%	98.0%
	Illness duration (years)	12.7 ± 9.7	12.9 ± 9.6	12.8 ± 9.7	13.8 ± 9.7	13.2 ± 9.7
	Restrictive subtype	59.6%	62.2%	60.4%	64.3%	60.1%
Data at admission	Weight (kg)	30.5 ± 6.4	30.0 ± 5.9	30.1 ± 6.0	29.7 ± 5.4	29.6 ± 5.4
	Body mass index (kg/m^2^)	12.2 ± 2.2	12.0 ± 2.1	12.1 ± 2.1	11.9 ± 2.0	11.9 ± 2.0
	Creatinine(mg/dl)	1.1 ± 1.3	1.0 ± 1.0	1.0 ± 1.0	1.0 ± 1.0	1.1 ± 1.2
	BUN/Cr ratio^1)^	30.0 ± 18.7	30.9 ± 19.6	30.6 ± 19.3	32.2 ± 19.7	32.1 ± 20.9
	Albumin(g/dl)	3.9 ± 0.7	3.9 ± 0.8	3.9 ± 0.7	3.9 ± 0.8	3.9 ± 0.8
	Phosphorus (mg/dl)	3.7 ± 1.4	3.7 ± 1.4	3.7 ± 1.4	3.7 ± 1.4	3.8 ± 1.5
	Potassium (mEq/l)	3.7 ± 1.0	3.6 ± 1.0	3.6 ± 1.0	3.6 ± 1.1	3.6 ± 1.0
	Magnesium(mg/dl)	2.3 ± 0.6	2.3 ± 0.7	2.3 ± 0.7	2.3 ± 0.7	2.3 ± 0.7
	Calcium(mg/dl) (adjusted Calcium)	9.0 ± 1.2 (9.2 ± 1.2)	9.0 ± 1.2 (9.2 ± 1.2)	9.0 ± 1.2 (9.2 ± 1.2)	9.0 ± 1.3 (9.2 ± 1.2)	8.9 ± 1.2 (9.2 ± 1.2)
Oral intake during the first 7 days	Intake (kcal/day)	1109 ± 669 **(167 ± 298 from enteral formulas)**	1100 ± 676 **(183 ± 312 from enteral formulas)**	1104 ± 679 **(180 ± 311 from enteral formulas)**	1214 ± 685 **(230 ± 339 from enteral formulas)**	1131 ± 685 **(188 ± 328 from enteral formulas)**
	Absolute oral electrolyte provision (mEq/day; phosphorus, mmol/day)	20.4 ± 12.2 for P^2)^ 45.1 ± 28.8 for K^3)^ 25.5 ± 26.9 for Mg^4)^ 20.7 ± 13.0 for Ca^5)^	20.3 ± 12.3 for P	45.3 ± 29.1 for K	28.6 ± 28.8 for Mg	21.2 ± 13.2 Ca
Oral intake during the first 24 h	Intake (kcal/day)	923 ± 703	907 ± 702	915 ± 705	1007 ± 710	928 ± 700
	Absolute oral electrolyte provision (mEq/day; phosphorus, mmol/day)	16.9 ± 13.1 for P 40.3 ± 33.1 for K 20.8 ± 25.6 for Mg 17.1 ± 13.6 for Ca	16.7 ± 13.1 for P	40.7 ± 33.6 for K	23.6 ± 28.0 for Mg	17.3 ± 13.5 for Ca
Parenteral intake during the first 7 days	Intake (kcal/day)	334 ± 360 (Dextrose 81.2%, amino acids 15.0%, lipids 3.8%)	377 ± 369 (Dextrose 80.9%. amino acids 15.1%, lipids 4.0%)	367 ± 364 (Dextrose 80.9%. amino acids 15.2%, lipids 3.9%)	380 ± 391 (Dextrose 80.7%. amino acids 14.6%, lipids 4.6%)	372 ± 381 (Dextrose 82.5%. amino acids 14.7%, lipids 2.7%)
	Parenteral electrolyte provision (mEq/day; phosphorus, mmol/day)	8.4 ± 11.2 for P 17.7 ± 19.0 for K 4.4 ± 6.4 for Mg 1.8 ± 2.5 for Ca	9.9 ± 11.6 for P	19.9 ± 19.3 for K	5.3 ± 7.3 for Mg	1.6 ± 2.4 for Ca
Parenteral intake during the first 24 h	Intake (kcal/day)	251 ± 324	276 ± 334	273 ± 332	283 ± 357	254 ± 329
	Parenteral electrolyte provision (mEq/day; phosphorus, mmol/day)	8.2 ± 12.6 for P 14.5 ± 16.9 for K 4.0 ± 7.5 for Mg 1.6 ± 2.8 for Ca	9.2 ± 13.1 for P	15.7 ± 17.3 for K	5.0 ± 8.7 for Mg	1.6 ± 2.8 for Ca
Parenteral electrolyte intake per calorie during the first 24 h relative to the first 7 days (%)		129.9% for P 109.0% for K 121.0% for Mg 118.3% for Ca	127.6%	106.1%	127.7%	146.5%
Parenteral electrolyte provision per calorie relative to oral provision (%) †	During the first 7 days (%)	136.7% for P 130.3% for K 57.3% for Mg 28.9% for Ca	141.5%	132.1%	58.7%	33.6%
	During the first 24 h (%)	178.4% for P 132.3% for K 70.7% for Mg 34.4% for Ca	177.5%	129.3%	75.4%	33.8%
Weight at discharge (kg)		33.9 ± 6.4	33.9 ± 6.3	33.9 ± 6.3	33.9 ± 6.2	33.6 ± 6.3
Nadir electrolyte level (nadir hospital day) Decline from admission (%)	Phosphorus (mg/dl) (nadir hospital day) Decline (%)	2.6 ± 0.8 (5.1 ± 4.6) -29.7%	2.6 ± 0.8 (5.1 ± 4.5) -29.7%	-	-	-
	Potassium (mEq/l) (nadir hospital day) Decline (%)	3.1 ± 0.7 (4.1 ± 4.3) -16.2%	-	3.1 ± 0.7 (4.1 ± 4.3) -13.9%	-	-
	Magnesium (mg/dl) (nadir hospital day) Decline (%)	1.8 ± 0.3 (6.4 ± 5.2) -21.7%	-	-	1.8 ± 0.3 (6.4 ± 5.2) -21.7%	-
	Calcium (mg/dl) (nadir hospital day) Decline (%)	8.0 ± 1.0 (5.9 ± 4.9) -10.1%	-	-	-	8.0 ± 0.9 (5.9 ± 4.7) -10.1%

Data are presented for the full admission cohort (leftmost column) and for the subsamples included in the nadir and percent-decrease analyses for each electrolyte (subsequent columns). Parenteral electrolyte provision per calorie during both the first 24 h and the first 7 hospital days was generally higher than oral provision for all electrolytes, after accounting for reported gastrointestinal absorption rates of orally administered electrolytes [see the note]

Data are expressed as mean ± standard deviation. (1) blood urea nitrogen/creatinine ratio; (2) phosphorus; (3) potassium; (4) magnesium; (5) calcium; † Reported gastrointestinal absorption rates of orally administered electrolytes from the literature: phosphorus 60–70%, potassium ≈ 90%, magnesium 30–50%, calcium 20–40% [57–69]

**Table 2:** Simple correlation analysis between electrolyte levels and calorie intake or electrolyte provision (unadjusted)

Cohort	Exposure variable	Outcome	Phosphorus	Potassium	Magnesium	Calcium
All participants (n = 172 for P^1);^ 182 for K^2);^ *140 for Mg*^3);^ 148 for Ca^4)^*)*	Total calorie (first 7 days)	Nadir level	p < 0.01** **r = -0.22**	p = 0.03* **r = 0.17**	p = 0.02* **r = -0.21**	p = 0.95 r = 0.00
		Percent decrease	p = 0.42 r = 0.06	p = 0.67 r = 0.03	p = 0.02* **r = 0.21**	p = 0.98 r = -0.00
	Electrolyte provision (adjusted) (first 24 h)	Nadir level	p = 0.19 r = -0.10	p < 0.001** **r = -0.38**	p = 0.57 r = 0.05	p = 0.26 r = 0.09
		Percent decrease	p = 0.97 r = -0.00	p = 0.07 r = -0.14	p = 0.28 r = -0.10	p = 0.02* **r = -0.20**
Participants with oral intake (n = 68 for P; 86 for K; 65 for Mg; 66 for Ca)	Oral calorie (first 7 days)	Nadir level	p = 0.85 r = 0.02	p = 0.84 r = 0.02	p = 0.02* **r = -0.29**	p = 0.32 r = 0.12
		Percent decrease	p = 0.85 r = -0.02	p = 0.74 r = 0.04	p = 0.07 r = 0.24	p = 0.30 r = -0.13
	Oral electrolyte provision (adjusted) (first 24 h)	Nadir level	p = 0.89 r = -0.02	p = 0.03* **r = -0.24**	p = 0.22 r = 0.15	p = 0.19 r = 0.16
		Percent decrease	p = 0.88 r = -0.02	p = 0.22 r= -0.13	p = 0.27 r = 0.15	p = 0.88 r = 0.02
Participants with parenteral intake (n = 18 for P; 18 for K; 9 for Mg; 15 for Ca)	Parenteral calorie (first 7 days)	Nadir level	p = 0.087 r = -0.42	p = 0.65 r = -0.12	p = 0.07 r = -0.64	p = 0.04* **r = -0.54**
		Percent decrease	p = 0.97 r = 0.01	p = 0.17 r = 0.34	p = 0.66 r = -0.19	p = 0.13 r = 0.41
	Parenteral electrolyte provision (first 24 h)	Nadir level	p = 0.87 r = 0.04	p = 0.34 r = -0.24	p = 0.63 r = -0.19	p = 0.08 r = 0.46
		Percent decrease	p = 0.12 r = -0.38	p = 0.20 r = -0.32	p = 0.35 r = -0.38	p < 0.01** **r = -0.69**

Results are shown for the whole cohort (upper section), participants receiving calories predominantly via the oral route (middle section), and those receiving calories predominantly via the parenteral route (bottom section)

Boldface indicates statistically significant associations. (1) phosphorus; (2) potassium; (3) magnesium; (4) calcium; Caloric intake reflects the first 7 hospital days; electrolyte provision reflects the first 24 h after admission. These analyses were not adjusted for multiple comparisons. **p < 0.01; * *p* < 0.05

**Table 3:** Electrolyte levels: significant factors from multivariable mixed-effects models

	Phosphorus	Potassium	Magnesium	Calcium
Admission level	Creatinine**	BUN/Cr ratio**, Creatinine*, Binge-purge behavior*	Creatinine**, Albumin**	Creatinine**, Albumin**, BMI*
Nadir level	Creatinine**, BUN/Cr ratio^1)^*, BMI^2)^*, Phosphorus level at admission**	Creatinine**, BMI*, Potassium level at admission**	Parenteral dextrose**, Creatinine*, Magnesium level at admission**	Creatinine**, BMI**, Albumin*, Calcium provision*, Calcium level at admission**
Percent decrease	Creatinine**, BUN/Cr ratio*, Phosphorus level at admission**	Creatinine**, BMI*, BUN/Cr ratio*, Potassium level at admission**	Parenteral dextrose **, Creatinine*, Magnesium level at admission**	Parenteral dextrose*, Creatinine**, BMI**, Calcium provision*, Calcium level at admission**

(1) blood urea nitrogen/creatinine ratio; (2) body mass index. **p < 0.01; * *p* < 0.05 (after multiple-comparison adjustment)

**Table 4:** Regression coefficients indicating the direction of route-specific effects on electrolyte levels during refeeding

	Phosphorus		Potassium		Magnesium		Calcium	
Nadir/percent decrease	Nadir	Percent decrease	Nadir	Percent decrease	Nadir	Percent decrease	Nadir	Percent decrease
Regular diet	-1.14 × 10^− 4^	0.0342	2.45 × 10^− 4^	-0.0467	-2.14 × 10^− 3^	0.0521	4.00 × 10^− 3^	-0.0526
Enteral formulas	-5.38 × 10^− 3^	0.189	3.50 × 10^− 4^	-0.0193	-2.13 × 10^− 3^	-0.0235	4.66 × 10^− 4^	4.25 × 10^− 3^
Parenteral dextrose	-0.0116	0.366	-5.38 × 10^− 3^	0.126	-0.119**	0.468**	-0.0134	0.172*
Parenteral non-dextrose	9.85 × 10^− 3^	-0.391	0.0179	-0.397	0.0106	-0.537	-0.0100	0.0851

Higher parenteral dextrose caloric intake was associated with lower nadir magnesium levels, as well as with greater percent decreases in magnesium and calcium

Negative regression coefficients for nadir levels indicate associations with lower nadir electrolyte levels, whereas positive regression coefficients for percent decrease indicate associations with greater percent decreases. **p < 0.01; * *p* < 0.05 (after multiple-comparison adjustment)

**Table 5:** Phosphorus levels: multivariable mixed-effects model (unadjusted)

Item	Admission level			Nadir level			Percent decrease		
	Beta	Standard error	*P*	Beta	Standard error	*P*	Beta	Standard error	*P*
Regular diet	**-**	**-**	**-**	-1.14 × 10^− 4^	3.05 × 10^− 3^	0.970	0.0342	0.0752	0.650
Enteral formulas	**-**	**-**	**-**	-5.38 × 10^− 3^	4.92 × 10^− 3^	0.276	0.189	0.121	0.121
Parenteral dextrose	**-**	**-**	**-**	-1.16 × 10^− 2^	6.38 × 10^− 3^	0.0702	**0.366**	**0.157**	0.0212*
Parenteral non-dextrose	**-**	**-**	**-**	9.85 × 10^− 3^	0.0193	0.611	-0.391	0.476	0.413
Phosphorus provision †	**-**	**-**	**-**	-2.35	3.34	0.483	-24.4	82.4	0.739
Creatinine	**0.670**	**0.123**	< 0.001**	**-0.273**	**0.0645**	< 0.001**	**5.81**	**1.59**	< 0.001**
BUN/Cr ratio	0.0111	5.83 × 10^− 3^	0.0578	**-9.69 × 10** ^**− 3**^	**3.50** × 10^− 3^	< 0.01**	**0.237**	**0.0861**	< 0.01**
Body mass index	-0.0168	0.0522	0.748	**0.0878**	**0.0305**	0.0120*	-1.05	0.850	0.218
Albumin	0.411	0.1667	0.015*	0.0312	0.0947	0.743	-3.17	2.33	0.177
Phosphorus level at admission	**-**	**-**	**-**	**0.211**	**0.0489**	< 0.001**	**7.72**	**1.21**	< 0.001**
Age	-0.0130	0.0140	0.355	-0.0163	7.60 × 10^− 3^	0.0332*	0.250	0.187	0.184
Illness duration	0.0121	0.0156	0.440	0.0169	7.91 × 10^− 3^	0.0342*	-0.344	0.195	0.0801
Female sex	-0.444	0.595	0.457	0.318	0.435	0.465	-1.09	10.7	0.919
Binge-purge	-0.100	0.274	0.717	0.0741	0.131	0.573	1.20	3.24	0.710

Boldface indicates statistically significant associations. †Adjusted for intestinal absorption of orally ingested phosphorus (assumed absorption rate, 65%); no adjustment was applied to parenterally administered phosphorus. **p < 0.01; *p < 0.05. Models were not adjusted for multiple comparisons

**Table 6:** Potassium levels: multivariable mixed-effects model (unadjusted)

Item	Admission level			Nadir level			Percent decrease		
	Beta	Standard error	*P*	Beta	Standard error	*P*	Beta	Standard error	*P*
Regular diet	**-**	**-**	**-**	2.45 × 10^− 4^	1.94 × 10^− 3^	0.900	-0.0467	0.0450	0.301
Enteral formulas	**-**	**-**	**-**	3.50 × 10^− 4^	2.86 × 10^− 3^	0.903	-0.0193	0.0664	0.771
Parenteral dextrose	**-**	**-**	**-**	-5.38 × 10^− 3^	3.45 × 10^− 3^	0.122	0.126	0.0800	0.117
Parenteral non-dextrose	**-**	**-**	**-**	0.0179	0.0106	0.0937	-0.397	0.246	0.108
Potassium provision †	**-**	**-**	**-**	-0.883	0.710	0.216	6.08	16.4	0.712
Creatinine	**0.226**	**0.0740**	< 0.01**	**-0.195**	**0.0426**	< 0.001**	**3.09**	**0.991**	< 0.01**
BUN/Cr ratio	**0.0107**	**3.41 × 10** ^**− 3**^	< 0.01**	-5.35 × 10^− 3^	2.17 × 10^− 3^	0.0147	**0.134**	**0.0503**	< 0.01**
Body mass index	0.0703	0.0315	0.027*	**0.0593**	**0.0213**	< 0.01**	**-1.50**	**0.495**	< 0.01**
Albumin	0.120	0.100	0.232	0.0312	0.0590	0.598	-0.154	1.37	0.911
Potassium level at admission	**-**	**-**	**-**	**0.560**	**0.0485**	< 0.001**	**7.33**	**1.12**	< 0.001**
Age	-9.01 × 10^3^	9.38 × 10^− 3^	0.339	-9.39 × 10^− 3^	4.96 × 10^− 3^	0.0611	**0.279**	**0.115**	0.0177*
Illness duration	7.88 × 10^− 3^	0.0104	0451	0.0131	5.29 × 10^− 3^	0.0150*	**-0.306**	**0.123**	0.0151*
Female sex	-0.900	0.397	0.606	0.0144	0.241	0.952	0.0805	5.60	0.989
Binge-purge	**-0.527**	**0.187**	< 0.01**	-0.0879	1.02	0.391	0.715	2.38	0.764

Boldface indicates statistically significant associations. †Adjusted for intestinal absorption of orally ingested potassium (assumed absorption rate, 90%); no adjustment was applied to parenterally administered potassium. **p < 0.01; *p < 0.05. Models were not adjusted for multiple comparisons

**Table 7:** Magnesium levels: multivariable mixed-effects model (unadjusted)

Item	Admission level			Nadir level			Percent decrease		
	Beta	Standard error	*P*	Beta	Standard error	*P*	Beta	Standard error	*P*
Regular diet	**-**	**-**	**-**	-2.14 × 10^− 3^	1.51 × 10^− 3^	0.161	0.0521	0.0521	0.321
Enteral formulas				-2.13 × 10^− 3^	2.08 × 10^− 3^	0.309	-0.0235	0.0740	0.7451
Parenteral dextrose	**-**	**-**	**-**	**-0.0119**	**2.58 × 10** ^**− 3**^	< 0.001**	**0.468**	**0.0945**	< 0.001**
Parenteral non-dextrose	**-**	**-**	**-**	0.0106	7.56 × 10^− 3^	0.1666	-0.537	0.281	0.0589
Magnesium provision †	**-**	**-**	**-**	0.671	2.32	0.773	-84.3	82.9	0.311
Creatinine	**0.413**	**0.0628**	< 0.001**	**-0.112**	**0.0398**	< 0.01**	**4.10**	**1.28**	< 0.01**
BUN/Cr ratio	6.65 × 10^− 3^	2.63 × 10^− 3^	0.0127*	-7.71 × 10^− 4^	1.60 × 10^− 3^	0.632	0.0836	0.0567	0.144
Body mass index	-0.0175	0.0246	0.477	4.06 × 10^− 3^	0.0176	0.818	-0.225	0.606	0.712
Albumin	**0.294**	**0.0808**	< 0.001**	8.87 × 10^− 3^	0.0510	0.862	0.769	1.78	0.666
Magnesium level at admission	**-**	**-**	**-**	**0.228**	**0.0486**	< 0.001**	**16.0**	**1.70**	< 0.001**
Age	-2.75 × 10^3^	6.77 × 10^− 3^	0.686	9.11 × 10^− 4^	3.98 × 10^− 3^	0.820	-0.0791	0.133	0.555
Illness duration	1.34 × 10^− 4^	6.97 × 10^− 3^	0.985	1.78 × 10^− 3^	4.02 × 10^− 3^	0.660	-0.0649	0.133	0.629
Female sex‡	-0.211	0.408	0.606	-	-	-	**-**	**-**	**-**
Binge-purge	0.0728	0.128	0.507	0.0103	0.0780	0.895	0.712	2.50	0.778

Boldface indicates statistically significant associations. **p < 0.01; *p < 0.05. †Adjusted for intestinal absorption of orally ingested magnesium (assumed absorption rate, 40%); no adjustment was applied to parenterally administered magnesium. ‡Female sex was removed from nadir level and percent decrease analyses in the mixed-effects model because it was perfectly collinear with binge-purge status in this specific cohort, resulting in a rank-deficient fixed-effect design matrix. Models were not adjusted for multiple comparisons

**Table 8:** Calcium levels: multivariable mixed-effects model (unadjusted)

Item	Admission level			Nadir level			Percent decrease		
	Beta	Standard error	*P*	Beta	Standard error	*P*	Beta	Standard error	*P*
Regular diet	**-**	**-**	**-**	4.00 × 10^− 3^	2.58 × 10^− 3^	0.126	-0.0526	0.0267	0.0549
Enteral formulas	**-**	**-**	**-**	4.66 × 10^− 4^	3.89 × 10^− 3^	0.905	4.25 × 10^− 3^	0.0404	0.916
Parenteral dextrose	**-**	**-**	**-**	**-0.0134**	**5.71 × 10** ^**− 3**^	0.0202*	**0.172**	**0.0595**	< 0.01**
Parenteral non-dextrose	-	-	-	-0.0100	2.15 × 10^− 2^	0.642	0.0851	0.224	0.704
Calcium provision †	**-**	**-**	**-**	**0.377**	**0.144**	< 0.01**	**-0.0398**	**0.0149**	< 0.01**
Creatinine	**0.475**	**0.0788**	< 0.001**	**-0.363**	**0.0604**	< 0.001**	**2.58**	**0.627**	< 0.001**
BUN/Cr ratio	4.36 × 10^− 3^	3.72 × 10^− 3^	0.242	-4.10 × 10^− 3^	2.82 × 10^− 3^	0.150	0.0291	0.0292	0.322
Body mass index	**0.0905**	**0.0356**	0.0120*	**0.0976**	**0.0300**	< 0.01**	**-0.993**	**0.332**	< 0.01*
Albumin	**0.834**	**0.106**	< 0.001**	**0.275**	**0.0916**	< 0.01**	-1.79	0.950	0.0633
Calcium level at admission	**-**	**-**	**-**	**0.316**	**0.0610**	< 0.001**	**5.27**	**0.634**	< 0.001**
Age	0.0132	9.19 × 10^− 3^	0.155	1.70 × 10^− 3^	6.60 × 10^− 3^	0.798	8.36 × 10^− 3^	0.0684	0.903
Illness duration	-0.0131	9.96 × 10^− 3^	0.194	-3.40 × 10^− 4^	6.67 × 10^− 3^	0.959	-3.06 × 10^− 3^	0.0691	0.965
Female sex	0.695	0.413	0.0953	0.714	0.415	0.0884	-8.41	4.32	0.0543
Binge-purge	0.204	0.179	0.194	0.0566	0.119	0.639	-0.668	1.23	0.593

Boldface indicates statistically significant associations. †Adjusted for intestinal absorption of orally ingested calcium (assumed absorption rate, 30%); no adjustment was applied to parenterally administered calcium. **p < 0.01; *p < 0.05. Models were not adjusted for multiple comparisons

**Table 9:** Multilevel correlation coefficient among the four electrolytes

Electrolyte combination	Admission level	Nadir level	Percent decrease
Phosphorus and potassium	*r* = 0.15 *p* = 0.76	*r* = 0.28 *p* = 0.05	*r* = 0.25 *p* = 0.09
Magnesium and phosphorus	*r* = 0.41 ***p*** < 0.001**	*r* = 0.21 *p* = 0.43	*r* = 0.28 *p* = 0.06
Phosphorus and calcium	*r* = 0.21 *p* = 0.27	*r* = 0.46 ***p*** < 0.001**	*r* = 0.27 *p* = 0.07
Magnesium and potassium	*r* = -0.09 *p* = 1.0	*r* = 0.08 *p* = 1.0	*r* = 0.24 *p* = 0.09
Potassium and calcium	*r* = 0.20 *p* = 0.29	*r* = 0.29 ***p*** = 0.04*	*r* = 0.35 ***p*** < 0.01**
Magnesium and calcium	*r* = 0.30 ***p*** < 0.01**	*r* = 0.12 *p* = 1.0	*r* = 0.34 ***p*** < 0.01**

Correlation coefficients between magnesium and potassium and between magnesium and calcium were not prominent compared with other electrolyte pairs. All electrolyte pairs showed positive directional associations at nadir and for percent decreases, regardless of statistical significance

Boldface indicates statistically significant associations. **p < 0.01; *p < 0.05 (after multiple-comparison adjustment)

## Discussion

This study demonstrated that caloric intake from a regular diet, enteral formulas, and parenteral non-dextrose nutrition was not significantly associated with electrolyte levels during the refeeding period in individuals with anorexia nervosa. In contrast, higher parenteral dextrose caloric intake was significantly associated with lower nadir magnesium levels and greater percent decreases in magnesium and calcium, despite higher or comparable electrolyte provision per calorie than oral intake after accounting for gastrointestinal absorption rates of orally administered electrolytes and the higher carbohydrate proportion in parenteral nutrition. Moreover, regardless of statistical significance, higher parenteral dextrose intake alone showed a consistent directional tendency toward lower nadir electrolyte levels and greater percent decreases across all electrolytes.

Regarding other covariates, lower BMI, a higher BUN/Cr ratio, and higher creatinine levels were frequently associated with lower nadir electrolyte levels and greater percent decreases during refeeding. Correlation analyses among electrolytes showed that associations involving magnesium—specifically with potassium and calcium—were not prominent at nadir and for percent decreases compared with other electrolyte pairs, whereas all electrolyte pairs showed positive directional trends at nadir and for percent decreases, regardless of statistical significance. Together, these findings suggest that global, concurrent electrolyte shifts during refeeding outweigh electrolyte-specific interactions involving magnesium.

### Route-specific nutritional associations with refeeding-related electrolyte disturbances

Previous studies—although limited and not systematically designed—have reported conflicting findings regarding which nutritional route is associated with more severe electrolyte deficiencies during refeeding, with one implicating parenteral feeding [9] and the other enteral nutrition using enteral formulas [21]. Our findings are more consistent with the former, as reported by Diamanti et al. (2008) [9], than with the latter, reported by Zeki et al. (2011) [21]. There was no marked difference in electrolyte deficiencies between the regular diet and enteral formulas in our study, likely because both were administered via the oral route with intestinal digestion, and their macronutrient composition and electrolyte content per calorie did not differ substantially. This finding is consistent with previous research [11, 12], although those studies did not comprehensively examine detailed electrolyte abnormalities during refeeding.

Our results cannot be explained by inherently different—and sometimes lower—electrolyte content (e.g., magnesium or calcium) in standard parenteral formulations compared with food-based nutrition [24], because parenteral electrolyte provision per calorie was higher than or comparable to oral provision in our cohort after accounting for reported gastrointestinal absorption rates of orally ingested electrolytes [56–68] and the higher carbohydrate proportion in parenteral nutrition than in regular diets and enteral formulas in our cohort. Nor can these findings be attributed to reactive parenteral electrolyte supplementation based on serial blood test results, as electrolyte provision per calorie during the first 24 h after admission was already higher than or comparable to that during the subsequent seven hospital days, and electrolyte provision was included as an explanatory variable in the multivariable models.

Rather, these findings are best explained by route-dependent differences in the rate of intestinal glucose absorption and hepatic first-pass carbohydrate handling. The finding that parenteral non-dextrose nutrition did not influence electrolyte deficiencies further suggests that parenteral dextrose itself may be the primary driver of electrolyte deficiencies during refeeding. Oral feeding delivers glucose gradually through intestinal absorption into the portal circulation, allowing the liver to extract a substantial proportion during first pass and to transiently buffer it via glycogen storage and glycolytic metabolism. This hepatic buffering presumably attenuates abrupt systemic increases in glucose and insulin, thereby limiting insulin-mediated intracellular shifts of phosphate, potassium, magnesium, and calcium during refeeding [22]. In contrast, parenteral glucose nutrition largely bypasses the portal circulation and hepatic first-pass metabolism, exposing peripheral tissues to more rapid and pronounced insulin signaling and increasing vulnerability to declines in serum electrolytes [70, 71].

Regarding the gut, in addition to gradual intestinal absorption, gastrointestinal adaptive regulation of electrolyte absorption may further contribute to the observed route-dependent differences. The fractional absorption of orally consumed electrolytes varies widely—approximately 90% for potassium, 60–70% for phosphorus, 30–50% for magnesium, and 20–40% for calcium—and is influenced by intake amount, with higher fractional absorption at lower dietary intakes, particularly for magnesium, calcium, and phosphorus [57–69]. During the early refeeding period, when dietary intake remains relatively low, fractional absorption may therefore be enhanced, potentially mitigating declines in serum electrolyte levels. However, some patients with severe anorexia nervosa exhibit intestinal malabsorption itself [14], which may to some extent offset these adaptive increases in electrolyte absorption.

Consistent with the concept of physiological buffering via the enteral route, enteral nutrition has been associated with better glycemic control and lower insulin requirements than parenteral nutrition in patients with acute pancreatitis, suggesting fundamental differences in metabolic handling between the enteral and parenteral routes [72]. In addition, several earlier studies—independent of the anorexia nervosa context—have linked severe refeeding-related electrolyte deficiencies to concentrated caloric delivery via total parenteral nutrition in malnourished patients [16–19]. These findings support our study findings that higher parenteral dextrose nutrition is associated with electrolyte deficiencies during refeeding in patients with anorexia nervosa. On the contrary, Zeki et al. (2011) [21] attributed their observation of an association between oral feeding and electrolyte deficiencies, compared with parenteral feeding during refeeding, to incretin-mediated augmentation of insulin secretion, which occurs with oral but not parenteral feeding. However, it should be noted that their study did not focus on patients with anorexia nervosa or severe malnutrition. While incretin signaling may enhance pancreatic insulin release, oral feeding is simultaneously characterized by gradual intestinal absorption, potential adaptive gut responses, and hepatic first-pass glucose handling, all of which attenuate systemic glucose and insulin exposure. Our findings suggest that these gut–liver buffering effects—absent in parenteral nutrition—likely play a more important role in determining insulin-mediated electrolyte shifts during refeeding than incretin-mediated insulin release alone, particularly in patients with anorexia nervosa and severe malnutrition.

The proportion of carbohydrates in parenteral nutrition in our cohort was higher than that in the regular diet and enteral formulas, reaching 81%. Considering the potential effects of carbohydrate load on insulin secretion and the subsequent development of refeeding-related electrolyte deficiencies [13, 22, 23, 26], a greater use of lipids in parenteral nutrition might have been preferable. Such an approach could have mitigated the marked electrolyte deficiencies observed in our cohort, as only higher parenteral dextrose intake was associated with these abnormalities. In this context, the practice outside Japan, where parenteral nutrition generally contains a higher proportion of lipids and a lower proportion of carbohydrates [36], may be more physiologically appropriate.

### Other factors associated with electrolyte refeeding syndrome

The associations between electrolyte levels and BMI, the BUN/Cr ratio, and serum creatinine observed in our study were consistent with those reported previously [32, 33, 40, 43, 46, 47]. Consistent with previous reports [32, 33, 40, 43], lower BMI was associated with multiple electrolyte deficiencies in our study, including lower nadir phosphorus, potassium, and calcium levels; greater percent decreases in potassium and calcium; and lower calcium levels at admission. Similarly, as previously reported [32, 40], a higher BUN/Cr ratio was associated with lower nadir levels in phosphorus and greater percent decreases in phosphorus as well as in potassium in our study. These findings are consistent with dehydration and hemoconcentration at admission, followed by dilutional and metabolic changes as hydration is restored during refeeding.

Higher calcium provision during the first 24 h was associated with higher nadir calcium levels and smaller percent decreases in calcium, suggesting a potential protective effect of early calcium supplementation. This interpretation is supported by simple correlation analyses, in which greater parenteral calcium provision was associated with smaller percent decreases in calcium in both the entire cohort and the parenteral subgroup. In contrast, although this association was not significant in the multivariable mixed-effects regression model, simple correlation analyses showed that higher potassium provision during the first 24 h was associated with lower potassium nadir levels. This counterintuitive finding is likely explained by the early occurrence of potassium nadirs, which were already present at admission in 69 admissions (37.9%), predominantly among individuals with the binge–purge subtype. In these cases, potassium was administered early in response to pre-existing hypokalemia at admission, resulting in an apparent association between higher early potassium provision and lower potassium nadir levels.

In addition, higher electrolyte levels at admission were consistently associated with greater percent decreases in the corresponding electrolytes, likely reflecting larger insulin-mediated intracellular shifts during refeeding, as individuals with higher initial levels presumably have a larger extracellular pool available for cellular uptake during anabolic recovery.

One of the most notable findings of our study, aside from the comparison between route-specific feeding, was that higher serum creatinine levels at admission were associated with higher electrolyte levels at admission, but also with lower nadir levels and greater percent decreases across all electrolytes examined. The positive association between creatinine and electrolyte levels at admission is well established [46]. However, the finding that higher creatinine levels were associated with lower nadir electrolyte levels during refeeding may appear counterintuitive. Notably, a previous report identified renal dysfunction as one of the strongest risk factors for refeeding syndrome, including electrolyte deficiencies [47], which is consistent with our results. In addition, renal dysfunction has been associated with hypomagnesemia due to renal magnesium wasting, although this observation was derived from a population with alcohol use disorders [73]. Taken together, these findings suggest that impaired renal compensatory capacity—particularly reduced tubular reabsorption—may exacerbate electrolyte depletion during refeeding. Nonetheless, the precise mechanisms underlying this association require further investigation.

### Clinical implications

Our results underscore the need for heightened vigilance regarding potential electrolyte deficiencies during parenteral dextrose administration, which are often critically important for patients with severe malnutrition. Accordingly, clinicians should closely monitor electrolyte levels during refeeding, with particular attention during parenteral dextrose nutrition, while remaining vigilant for electrolyte declines even with oral intake. Prophylactic electrolyte supplementation has been suggested as a preventive strategy [26], particularly in individuals with low electrolyte levels at admission and additional risk factors identified in our study, such as low BMI.

### Limitations

We acknowledge several limitations that should be considered when generalizing our results. First, we did not perform a direct comparison between a cohort receiving oral intake only and one receiving parenteral nutrition only because nutritional routes were often combined in our cohort. In this context, we used multivariable regression analyses adjusted for BMI and biochemical markers of nutritional and clinical status (BUN/Cr ratio and albumin levels), allowing us to evaluate the independent effects of each nutritional route and component, separate from patients’ underlying physical condition. However, parenteral nutrition may have been preferentially administered to more severely ill patients, and residual confounding by indication cannot be entirely excluded. Nevertheless, the absence of an association for parenteral non-dextrose nutrition suggests that this bias alone is unlikely to explain the observed association with parenteral dextrose. Taken together, these analyses help clarify the association between higher parenteral dextrose nutrition and electrolyte deficiencies.

Second, although we made every effort to quantify caloric and electrolyte intake as accurately as possible, actual regular diet may have differed from the recorded values. Some inpatients consumed snacks, sweets, or other foods purchased from the hospital convenience store or brought from home by family members. These items contributed to oral caloric and electrolyte intake but were not included in our calculations because the amounts consumed and their electrolyte content could not be reliably monitored. Although patients likely consumed unrecorded snacks and sweets that are typically high in carbohydrates and low in electrolytes per calorie [74], we observed no significant association between regular diet caloric intake and electrolyte declines. Because inclusion of such intake would increase dextrose caloric exposure without a proportional increase in electrolyte intake, this limitation would be expected to bias analyses toward underestimating regular diet dextrose caloric intake and, if anything, make a true negative association more apparent. Therefore, the absence of an association suggests that regular diet caloric amount was not a dominant driver of electrolyte decline in our cohort, in contrast to the clearer risk signal observed with parenteral dextrose feeding. In addition, for the regular diet, macronutrient and electrolyte intake was not estimated based on the exact proportions of individual staple foods (e.g., rice, bread, or noodles) or side dishes consumed. Instead, caloric and electrolyte intake were estimated using average consumption ratios relative to the entire preset meal, and macronutrient intake (carbohydrate, protein, and fat) was not calculated separately. We were also unable to quantify the frequency or volume of vomiting in patients with binge–purge behaviors. However, binge–purge subtype was included as an explanatory variable to account for its potential impact on electrolyte levels. Vomiting itself is unlikely to alter the proportion of electrolyte provision relative to caloric intake, because vomited contents originate primarily from the stomach, whereas nutrient and electrolyte absorption occurs mainly in the intestine [75, 76]. Despite these limitations, to our knowledge, this is the first study to attempt a comprehensive quantification of caloric and electrolyte intake from both route-specific nutrition in the context of refeeding.

Third, exact nadir electrolyte levels may have been missed because, although blood tests were performed frequently after admission, they were not conducted daily. Fourth, as described earlier, electrolyte provision per calorie from oral and parenteral routes was combined into a single explanatory variable in the multivariable mixed-effects model and therefore could not be evaluated separately by route. Fifth, missing data were present, particularly for magnesium levels. Sixth, parenteral nutrition was not stratified by administration pattern (continuous 24-hour vs. intermittent infusion), which may influence the magnitude of insulin surges during refeeding. Seventh, gastrointestinal electrolyte absorption rates and hepatic first-pass glucose handling were not directly evaluated in this study. Future studies should specifically examine how different macronutrients and parenteral nutrition administration patterns influence insulin secretion and electrolyte dynamics, as well as how liver function modulates electrolyte abnormalities during the refeeding period. Lastly, this was a single-center study; therefore, multicenter studies incorporating facilities with differing caloric prescriptions and electrolyte supplementation protocols are needed to confirm the generalizability of our findings.

## Conclusions

Our study found that higher parenteral dextrose nutrition during the refeeding period was associated with electrolyte deficiencies in patients with anorexia nervosa, underscoring the need for heightened vigilance for electrolyte disturbances, particularly during parenteral feeding, which is often essential for many patients with severe malnutrition.

## Data Availability

Data are available upon request. For further details, please contact the corresponding author, M.F.

## References

[1] Golden NH, Keane-Miller C, Sainani KL, Kapphahn CJ. Higher caloric intake in hospitalized adolescents with anorexia nervosa is associated with reduced length of stay and no increased rate of refeeding syndrome. J Adolesc Health. 2013;53:573–8. 10.1016/j.jadohealth.2013.05.014. 23830088 10.1016/j.jadohealth.2013.05.014

[2] Matthews K, Hill J, Jeffrey S, Patterson S, Davis A, Ward W, Palmer M, Capra S. A Higher-Calorie Refeeding Protocol Does Not Increase Adverse Outcomes in Adult Patients with Eating Disorders. J Acad Nutr Diet. 2018;118:1450–63. 10.1016/j.jand.2018.01.023. 29656932 10.1016/j.jand.2018.01.023

[3] Schlapfer L, Fujimoto A, Gettis M. Impact of caloric prescriptions and degree of malnutrition on incidence of refeeding syndrome and clinical outcomes in patients with eating disorders: A retrospective review. Nutr Clin Pract. 2022;37:459–69. 10.1002/ncp.10792. 34751947 10.1002/ncp.10792

[4] Ibrahim N, Barruchet A, Moro MR, Blanchet C. Severe neutropenia in an anorexic adolescent girl: a stigma of underfeeding syndrome? Eat Weight Disord. 2021;26:1271–5. 10.1007/s40519-020-01016-0. 32978756 10.1007/s40519-020-01016-0

[5] Garber AK, Cheng J, Accurso EC, Adams SH, Buckelew SM, Kapphahn CJ, Kreiter A, Le Grange D, Machen VI, Moscicki AB, Sy A, Wilson L, Golden NH. Short-term Outcomes of the Study of Refeeding to Optimize Inpatient Gains for Patients With Anorexia Nervosa: A Multicenter Randomized Clinical Trial. JAMA Pediatr. 2021;1:175:19–27. 10.1001/jamapediatrics.2020.3359. 10.1001/jamapediatrics.2020.3359 PMC7573797 33074282

[6] Kohn MR, Golden NH. Management of the malnourished patient: it’s now time to revise the guidelines. J Eat Disord. 2022;10:56. 10.1186/s40337-022-00539-4. 35440063 10.1186/s40337-022-00539-4 PMC9019959

[7] Robinson P, Rhys Jones W. MARSIPAN: management of really sick patients with anorexia nervosa. BJPsych Adv. 2018;24:20–32. 10.1192/bja.2017.2.

[8] Ibrahim N, Barruchet A, Moro MR, Blanchet C. Severe neutropenia in an anorexic adolescent girl: a stigma of underfeeding syndrome? Eat Weight Disord. 2021;26(4):1271–5. 10.1007/s40519-020-01016-0. 32978756 10.1007/s40519-020-01016-0

[9] Diamanti A, Basso MS, Castro M, Bianco G, Ciacco E, Calce A, Caramadre AM, Noto C, Gambarara M. Clinical efficacy and safety of parenteral nutrition in adolescent girls with anorexia nervosa. J Adolesc Health. 2008;42:111–8. 10.1016/j.jadohealth.2007.09.024. 18207088 10.1016/j.jadohealth.2007.09.024

[10] Michihata N, Matsui H, Fushimi K, Yasunaga H. Comparison between enteral nutrition and intravenous hyperalimentation in patients with eating disorders: results from the Japanese diagnosis procedure combination database. Eat Weight Disord. 2014;19:473–8. 10.1007/s40519-014-0147-y. 25150426 10.1007/s40519-014-0147-y

[11] Rizzo SM, Douglas JW, Lawrence JC. Enteral Nutrition via Nasogastric Tube for Refeeding Patients With Anorexia Nervosa: A Systematic Review. Nutr Clin Pract. 2019;34:359–70. 10.1002/ncp.10187. 30070730 10.1002/ncp.10187

[12] Martini M, Longo P, Di Benedetto C, Delsedime N, Panero M, Abbate-Daga G, Toppino F. Nasogastric Tube Feeding in Anorexia Nervosa: A Propensity Score-Matched Analysis on Clinical Efficacy and Treatment Satisfaction. Nutrients. 2024;16:1664. 10.3390/nu16111664. 38892597 10.3390/nu16111664 PMC11174568

[13] Garber AK, Sawyer SM, Golden NH, Guarda AS, Katzman DK, Kohn MR, Le Grange D, Madden S, Whitelaw M, Redgrave GW. A systematic review of approaches to refeeding in patients with anorexia nervosa. Int J Eat Disord. 2016;49:293–310. 10.1002/eat.22482. 26661289 10.1002/eat.22482 PMC6193754

[14] Malczyk Ż, Oświęcimska JM. Gastrointestinal complications and refeeding guidelines in patients with anorexia nervosa. Psychiatr Pol. 2017;51:219–29. 10.12740/PP/65274. English, Polish. 28581533 10.12740/PP/65274

[15] Prabhakaran S, Doraiswamy VA, Nagaraja V, Cipolla J, Ofurum U, Evans DC, Lindsey DE, Seamon MJ, Kavuturu S, Gerlach AT, Jaik NP, Eiferman DS, Papadimos TJ, Adolph MD, Cook CH. Stawickiet SPA Nasoenteric Tube Complications. Scand J Surg. 2012;101(3):147–55. 10.1177/145749691210100302. 22968236 10.1177/145749691210100302

[16] Weinsier RL, Krumdieck CL. Death resulting from overzealous total parenteral nutrition: the refeeding syndrome revisited. Am J Clin Nutr. 1981;34(3):393–9. 10.1093/ajcn/34.3.393. 6782855 10.1093/ajcn/34.3.393

[17] Solomon SM, Kirby DF. The refeeding syndrome: a review. JPEN J Parenter Enter Nutr. 1990;14:90–7. 10.1177/014860719001400190. 10.1177/014860719001400190 2109122

[18] Veldscholte K, Veen MAN, Eveleens RD, de Jonge RCJ, Vanhorebeek I, Gunst J, Casaer MP, Wouters PJ, Guerra GG, Van den Berghe G, Joosten KFM, Verbruggen SCAT. Early hypophosphatemia in critically ill children and the effect of parenteral nutrition: A secondary analysis of the PEPaNIC RCT. Clin Nutr. 2022;41(11):2500–8. 10.1016/j.clnu.2022.09.001. 36219978 10.1016/j.clnu.2022.09.001

[19] Russo Hortencio TD, Springer AMM, Silveira LR, Golucci APBS, Filho RMS, Nogueira RJN. Moderate-severe hypophosphatemia, hypomagnesemia, and hypokalemia disturbances in critically ill patients receiving total parenteral nutrition therapy. Clin Nutr ESPEN. 2025;68:55–61. 10.1016/j.clnesp.2025.04.023. 40311927 10.1016/j.clnesp.2025.04.023

[20] Borriello R, Esposto G, Ainora ME, Podagrosi G, Ferrone G, Mignini I, Galasso L, Gasbarrini A, Zocco MA. Understanding Refeeding Syndrome in Critically Ill Patients: A Narrative Review. Nutrients. 2025;17(11):1866. 10.3390/nu17111866. 40507135 10.3390/nu17111866 PMC12157793

[21] Zeki S, Culkin A, Gabe SM, Nightingale JM. Refeeding hypophosphataemia is more common in enteral than parenteral feeding in adult in patients. Clin Nutr. 2011;30:365–8. 10.1016/j.clnu.2010.12.001. 21256638 10.1016/j.clnu.2010.12.001

[22] Mehanna HM, Moledina J, Travis J. Refeeding syndrome: what it is, and how to prevent and treat it. BMJ. 2008;336:1495–8. 10.1136/bmj.a301. 18583681 10.1136/bmj.a301 PMC2440847

[23] Berlana D. Parenteral Nutrition Overview. Nutrients. 2022;14(21):4480. 10.3390/nu14214480. 36364743 10.3390/nu14214480 PMC9659055

[24] Braga CB, Ferreira IM, Marchini JS, Cunha SF. Copper and magnesium deficiencies in patients with short bowel syndrome receiving parenteral nutrition or oral feeding. Arq Gastroenterol. 2015;52:94–9. 10.1590/S0004-28032015000200004. 26039825 10.1590/S0004-28032015000200004

[25] Kraft MD, Btaiche IF, Sacks GS, Kudsk KA. Treatment of electrolyte disorders in adult patients in the intensive care unit. Am J Health Syst Pharm. 2005;62:1663–82. 10.2146/ajhp040300. 16085929 10.2146/ajhp040300

[26] Friedli N, Stanga Z, Culkin A, Crook M, Laviano A, Sobotka L, Kressig RW, Kondrup J, Mueller B, Schuetz P. Management and prevention of refeeding syndrome in medical inpatients: An evidence-based and consensus-supported algorithm. Nutrition. 2018;47:13–20. 10.1016/j.nut.2017.09.007. 29429529 10.1016/j.nut.2017.09.007

[27] World Health Organization. International statistical classification of diseases and related health problems, 10th revision. 5th ed. Geneva: World Health Organization; 2016.

[28] Schalla MA, Stengel A. Gastrointestinal alterations in anorexia nervosa - A systematic review. Eur Eat Disord Rev. 2019;27:447–61. 10.1002/erv.2679. 31062912 10.1002/erv.2679

[29] Rząd Z, Rog J, Kajka N, Szewczyk P, Krukow P, Karakuła-Juchnowicz H. The efficacy of transcranial direct current stimulation in the treatment of anorexia nervosa: a randomized double-blind clinical trial. Front Psychiatry. 2024;15:1284675. 10.3389/fpsyt.2024.1284675. 38757134 10.3389/fpsyt.2024.1284675 PMC11096801

[30] Funayama M, Takata T, Koreki A, Ogino S, Mimura M. Catatonic Stupor in Schizophrenic Disorders and Subsequent Medical Complications and Mortality. Psychosom Med. 2018;80(4):370–6. 10.1097/PSY.0000000000000574. 29521882 10.1097/PSY.0000000000000574 PMC5959200

[31] Abbate-Daga G, Amianto F, Delsedime N, De-Bacco C, Fassino S. Resistance to treatment and change in anorexia nervosa: a clinical overview. BMC Psychiatry. 2013;13:294. 10.1186/1471-244X-13-294. 24199620 10.1186/1471-244X-13-294 PMC3879222

[32] Funayama M, Mimura Y, Takata T, Koreki A, Ogino S, Kurose S. Body mass index and blood urea nitrogen to creatinine ratio predicts refeeding hypophosphatemia of anorexia nervosa patients with severe malnutrition. J Eat Disord. 2021a;9:1. 10.1186/s40337-020-00356-7. 33407855 10.1186/s40337-020-00356-7 PMC7789160

[33] Funayama M, Mimura Y, Takata T, Koreki A, Ogino S, Kurose S, Shimizu Y. Hypokalemia in patients with anorexia nervosa during refeeding is associated with binge-purge behavior, lower body mass index, and hypoalbuminemia. J Eat Disord. 2021b;9:95. 10.1186/s40337-021-00452-2. 34362446 10.1186/s40337-021-00452-2 PMC8348865

[34] Funayama M, Koreki A, Mimura Y, Takata T, Ogino S, Kurose S, Shimizu Y, Kudo S. Restrictive type and infectious complications might predict nadir hematological values among individuals with anorexia nervosa during the refeeding period: a retrospective study. J Eat Disord. 2022;10:64. 10.1186/s40337-022-00586-x. 35513879 10.1186/s40337-022-00586-x PMC9074196

[35] Gibson D, Watters A, Cost J, Mascolo M, Mehler PS. Extreme anorexia nervosa: medical findings, outcomes, and inferences from a retrospective cohort. J Eat Disord. 2020;8:25. 10.1186/s40337-020-00303-6. 32582446 10.1186/s40337-020-00303-6 PMC7310519

[36] Takagi K, Murotani K, Kamoshita S, Kuroda A. Clinical impact of lipid injectable emulsion in internal medicine inpatients exclusively receiving parenteral nutrition: a propensity score matching analysis from a Japanese medical claims database. BMC Med. 2022;20(1):371. 10.1186/s12916-022-02568-x. 36289527 10.1186/s12916-022-02568-x PMC9608912

[37] da Silva JSV, Seres DS, Sabino K, Adams SC, Berdahl GJ, Citty SW, Cober MP, Evans DC, Greaves JR, Gura KM, Michalski A, Plogsted S, Sacks GS, Tucker AM, Worthington P, Walker RN, Ayers P, American Society for Parenteral and Enteral Nutrition. Parenteral Nutrition Safety and Clinical Practice Committees,. ASPEN Consensus Recommendations for Refeeding Syndrome. Nutr Clin Pract. 2020;35:178–195. 10.1002/ncp.10474. Epub 2020 Mar 2. Erratum in: Nutr Clin Pract. 2020;35(3):584–585. doi: 10.1002/ncp.10491. 10.1002/ncp.10474 32115791

[38] Cederholm T, Bosaeus I. Malnutrition in Adults. N Engl J Med. 2024;391:155–65. 10.1056/NEJMra2212159. 38986059 10.1056/NEJMra2212159

[39] Gallagher D, Parker A, Samavat H, Zelig R. Prophylactic supplementation of phosphate, magnesium, and potassium for the prevention of refeeding syndrome in hospitalized individuals with anorexia nervosa. Nutr Clin Pract. 2022;37:328–43. 10.1002/ncp.10786. 34648201 10.1002/ncp.10786

[40] Kameoka N, Iga J, Tamura M, Tominaga T, Kubo H, Watanabe Y, Sumitani S, Tomotake M, Omori T. Risk factors for refeeding hypophosphatemia in Japanese inpatients with anorexia nervosa. Int J Eat Disord. 2016;49:402–6. 10.1002/eat.22472. 26446402 10.1002/eat.22472

[41] Swaminathan R. Magnesium metabolism and its disorders. Clin Biochem Rev. 2003;24:47–66. 18568054 PMC1855626

[42] Desgagnés N, King JA, Kline GA, Seiden-Long I, Leung AA. Use of Albumin-Adjusted Calcium Measurements in Clinical Practice. JAMA Netw Open 2025 2;8:e2455251. 10.1001/jamanetworkopen.2024.55251 10.1001/jamanetworkopen.2024.55251 PMC11751745 39836424

[43] Brown CA, Sabel AL, Gaudiani JL, Mehler PS. Predictors of hypophosphatemia during refeeding of patients with severe anorexia nervosa. Int J Eat Disord. 2015;48:898–904. 10.1002/eat.22406. 25846384 10.1002/eat.22406

[44] Hosten AO. Chapter 193 BUN and Creatinine. In: Walker HK, Hall WD, Hurst JW, editors. Clinical methods: the history, physical, and laboratory examinations. 3rd ed. Boston: Butterworths; 1990. 21250045

[45] Caregoro L, Di Pascoli L, Favoro A, Nardi M, Santonastaso P. Sodium depletion and hemoconcentration: overlooked complications in patients with anorexia nervosa? Nutrition. 2005;21:438–45. 10.1016/j.nut. 2004. 08. 022. 10.1016/j.nut.2004.08.022 15811763

[46] Dhondup T, Qian Q. Acid-Base and Electrolyte Disorders in Patients with and without Chronic Kidney Disease: An Update. Kidney Dis (Basel). 2017;3:136–48. 10.1159/000479968. 29344508 10.1159/000479968 PMC5757582

[47] Adika E, Jia R, Li J, Seres D, Freedberg DE. Evaluation of the ASPEN guidelines for refeeding syndrome among hospitalized patients receiving enteral nutrition: A retrospective cohort study. JPEN J Parenter Enter Nutr. 2022;46:1859–66. 10.1002/jpen.2368. 10.1002/jpen.2368 PMC9464262 35274317

[48] Arampatzis S, Funk GC, Leichtle AB, Fiedler GM, Schwarz C, Zimmermann H, Exadaktylos AK, Lindner G. Impact of diuretic therapy-associated electrolyte disorders present on admission to the emergency department: a cross-sectional analysis. BMC Med. 2013;11:83. 10.1186/1741-7015-11-83. 23531202 10.1186/1741-7015-11-83 PMC3621479

[49] Kieboom BCT, Zietse R, Ikram MA, Hoorn EJ, Stricker BH. Thiazide but not loop diuretics is associated with hypomagnesaemia in the general population. Pharmacoepidemiol Drug Saf. 2018;27(11):1166–73. 10.1002/pds.4636. 30095199 10.1002/pds.4636

[50] Grieff M, Bushinsky DA. Diuretics and disorders of calcium homeostasis. Semin Nephrol. 2011;31(6):535–41. 10.1016/j.semnephrol.2011.09.008. 22099510 10.1016/j.semnephrol.2011.09.008

[51] LePane C, Peleman R, Kinzie J. Chronic diarrhea with hyperchloremic acidosis and hypokalemia. Gastroenterology. 2012;142(1):e22–3. 10.1053/j.gastro.2011.01.065. 22107713 10.1053/j.gastro.2011.01.065

[52] Floris M, Angioi A, Lepori N, Piras D, Cabiddu G, Pani A, Rosner MH. The Clinical Spectrum of Acquired Hypomagnesemia: From Etiology to Therapeutic Approaches. Biomedicines. 2025;13(8):1862. 10.3390/biomedicines13081862. 40868117 10.3390/biomedicines13081862 PMC12383300

[53] Park CH, Kim EH, Roh YH, Kim HY, Lee SK. The association between the use of proton pump inhibitors and the risk of hypomagnesemia: a systematic review and meta-analysis. PLoS ONE. 2014;9(11):e112558. 10.1371/journal.pone.0112558. 25394217 10.1371/journal.pone.0112558 PMC4230950

[54] Huang CL, Kuo E. Mechanism of hypokalemia in magnesium deficiency. J Am Soc Nephrol. 2007;18:2649–52. 10.1681/ASN.2007070792. 17804670 10.1681/ASN.2007070792

[55] Zamani M, Haghighat N. The Effects of Magnesium Supplementation on Serum Magnesium and Calcium Concentration in Patients With Type 2 Diabetes: A Systematic Review and Meta-Analysis of Randomized Controlled Trials. Clin Nutr Res. 2022;11:133–45. 10.7762/cnr.2022.11.2.133. 35559000 10.7762/cnr.2022.11.2.133 PMC9065397

[56] Morino K, Kondo K, Tanaka S, Nishida Y, Nakae S, Yamada Y, Ugi S, Fuse K, Miyazawa I, Ohi A, Nishida K, Kurihara M, Sasaki M, Ebine N, Sasaki S, Katsukawa F, Hiroshi M. Total energy expenditure is comparable between patients with and without diabetes mellitus: Clinical Evaluation of Energy Requirements in Patients with Diabetes Mellitus (CLEVER-DM) Study. BMJ Open Diabetes Res Care. 2019;7:e000648. doi: 10. 1136/bmjdrc- 2019- 00064 8eCol lecti on2019. 31114702 10.1136/bmjdrc-2019-000648 PMC6501857

[57] Macdonald-Clarke CJ, Martin BR, McCabe LD, McCabe GP, Lachcik PJ, Wastney ME, Weaver CM. Bioavailability of potassium from potatoes and potassium gluconate: a randomized dose-response trial. Am J Clin Nutr. 2016;104:346–53. 10.3945/ajcn.115.127225. 27413123 10.3945/ajcn.115.127225

[58] Hinderling PH. The pharmacokinetics of potassium in humans is unusual. J Clin Pharmacol. 2016;56:1212–20. 10.1002/jcph.713. 26854277 10.1002/jcph.713

[59] Peacock M. Phosphate metabolism in health and disease. Calcif Tissue Int. 2021;108:3–15. 10.1007/s00223-020-00686-3. 32266417 10.1007/s00223-020-00686-3

[60] Wagner CA. The basics of phosphate metabolism. Nephrol Dial Transpl. 2024;39:190–201. 10.1093/ndt/gfad188. 10.1093/ndt/gfad188 PMC10828206 37660247

[61] Hill Gallant KM, Vorland CJ. Intestinal phosphorus absorption: recent findings in translational and clinical research. Curr Opin Nephrol Hypertens. 2021;30:404–10. 10.1097/MNH.0000000000000719. 34027902 10.1097/MNH.0000000000000719 PMC8153371

[62] Hernando N, Wagner CA. Mechanisms and regulation of intestinal phosphate absorption. Compr Physiol. 2018;8:1065–90. 10.1002/cphy.c170024. 29978897 10.1002/cphy.c170024

[63] Stremke ER, Wiese GN, Moe SM, Wastney ME, Moorthi RN, Hill Gallant KMH. Intestinal phosphorus absorption in moderate CKD and healthy adults determined using a radioisotopic tracer. J Am Soc Nephrol. 2021;32:2057–69. 10.1681/ASN.2020091340. 34244325 10.1681/ASN.2020091340 PMC8455256

[64] Touyz RM, de Baaij JHF, Hoenderop JGJ. Magnesium disorders. N Engl J Med. 2024;390:1998–2009. 10.1056/NEJMra1510603. 38838313 10.1056/NEJMra1510603

[65] Fine KD, Santa Ana CA, Porter JL, Fordtran JS. Intestinal absorption of magnesium from food and supplements. J Clin Invest. 1991;88:396–402. 10.1172/JCI115317. 1864954 10.1172/JCI115317 PMC295344

[66] Chamniansawat S, Suksridechacin N, Thongon N. Current opinion on the regulation of small intestinal magnesium absorption. World J Gastroenterol. 2023;29:332–42. 10.3748/wjg.v29.i2.332. 36687126 10.3748/wjg.v29.i2.332 PMC9846944

[67] Areco VA, Kohan R, Talamoni G, Tolosa de Talamoni NG, Peralta López ME. Intestinal Ca absorption revisited: a molecular and clinical approach. World J Gastroenterol. 2020;26:3344–64. 10.3748/wjg.v26.i24.3344. 32655262 10.3748/wjg.v26.i24.3344 PMC7327788

[68] Wongdee K, Chanpaisaeng K, Teerapornpuntakit J, Charoenphandhu N. Intestinal calcium absorption. Compr Physiol. 2021;11:2047–73. 10.1002/cphy.c200014. 34058017 10.1002/cphy.c200014

[69] Gumz ML, Rabinowitz L, Wingo CS. An integrated view of potassium homeostasis. N Engl J Med. 2015;373:60–72. 10.1056/NEJMra1313341. 26132942 10.1056/NEJMra1313341 PMC5675534

[70] Ishida T, Chap Z, Chou J, Lewis R, Hartley C, Entman M, Field JB. Differential effects of oral, peripheral intravenous, and intraportal glucose on hepatic glucose uptake and insulin and glucagon extraction in conscious dogs. J Clin Invest. 1983;72:590–601. 10.1172/JCI111007. 6348094 10.1172/JCI111007 PMC1129217

[71] Dimitriadis GD, Maratou E, Kountouri A, Board M, Lambadiari V. Regulation of Postabsorptive and Postprandial Glucose Metabolism by Insulin-Dependent and Insulin-Independent Mechanisms: An Integrative Approach. Nutrients. 2021;13:159. 10.3390/nu13010159. 33419065 10.3390/nu13010159 PMC7825450

[72] Petrov MS, Zagainov VE. Influence of enteral versus parenteral nutrition on blood glucose control in acute pancreatitis: a systematic review. Clin Nutr. 2007;26:514–23. 10.1016/j.clnu.2007.04.009. 17559987 10.1016/j.clnu.2007.04.009

[73] Hernández-Rubio A, Sanvisens A, Barbier-Torres L, Blanes R, Miquel L, Torrens M, Rubio G, Bolao F, Zuluaga P, Fuster D, Rodríguez de Fonseca F, Farré M, Muga R, CohRTA. Associations of hypomagnesemia in patients seeking a first treatment of alcohol use disorder. Drug Alcohol Depend. 2023;245:109822. 10.1016/j.drugalcdep.2023.109822. 36893509 10.1016/j.drugalcdep.2023.109822

[74] Schiefermeier-Mach N, Egg S, Erler J, Hasenegger V, Rust P, König J, Purtscher AE. Electrolyte Intake and Major Food Sources of Sodium, Potassium, Calcium and Magnesium among a Population in Western Austria. Nutrients. 2020;12:1956. 10.3390/nu12071956 10.3390/nu12071956 PMC7400604 32630029

[75] Kiela PR, Ghishan FK. Physiology of Intestinal Absorption and Secretion. Best Pract Res Clin Gastroenterol. 2016;30:145–59. 10.1016/j.bpg.2016.02.007. 27086882 10.1016/j.bpg.2016.02.007 PMC4956471

[76] Basile EJ, Shukla K, Launico MV, Physiology et al. Nutrient Absorption. [Updated 2025 Dec 1]. In: StatPearls [Internet]. Treasure Island (FL): StatPearls Publishing; 2025 Jan-. Available from:https://www.ncbi.nlm.nih.gov/books/NBK597379/?utm_source=chatgpt.com (Last Update: December 1, 2025).

